# Supplementary figures and images for: Phosphoproteome Dynamics of Streptomyces rimosus during Submerged Growth and Antibiotic Production
Source: mSystems. 2022 Sep 12;7(5):e00199-22. doi: 10.1128/msystems.00199-22 (PMC9600765; doi:10.1128/msystems.00199-22)

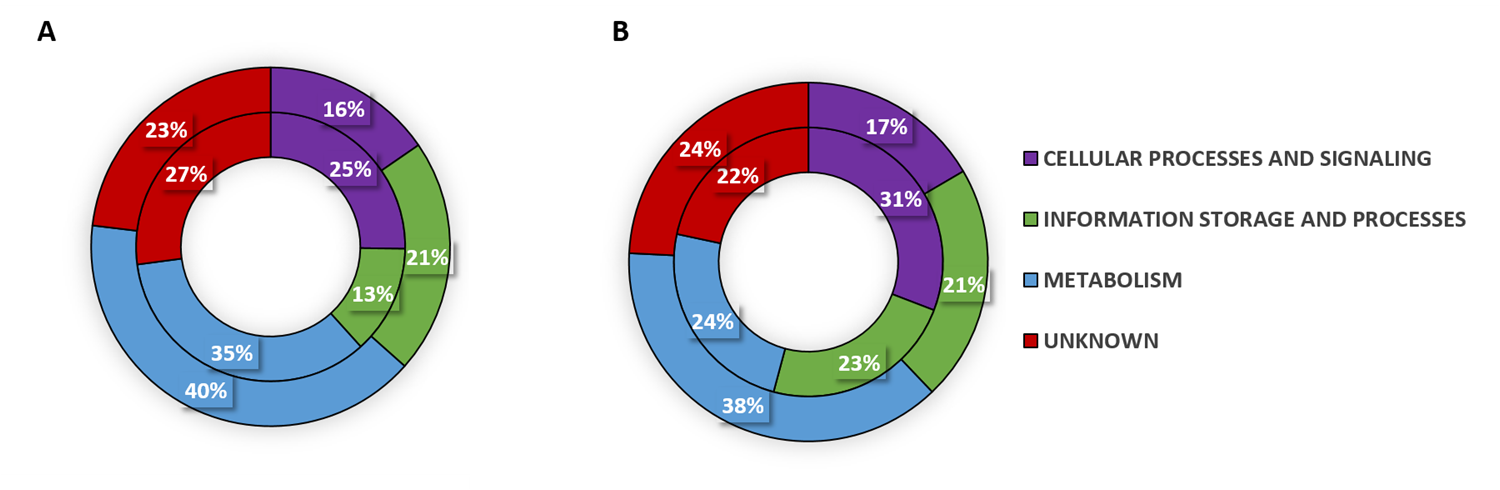

Supplement: FIG S1 [file msystems.00199-22-s0001.tif]

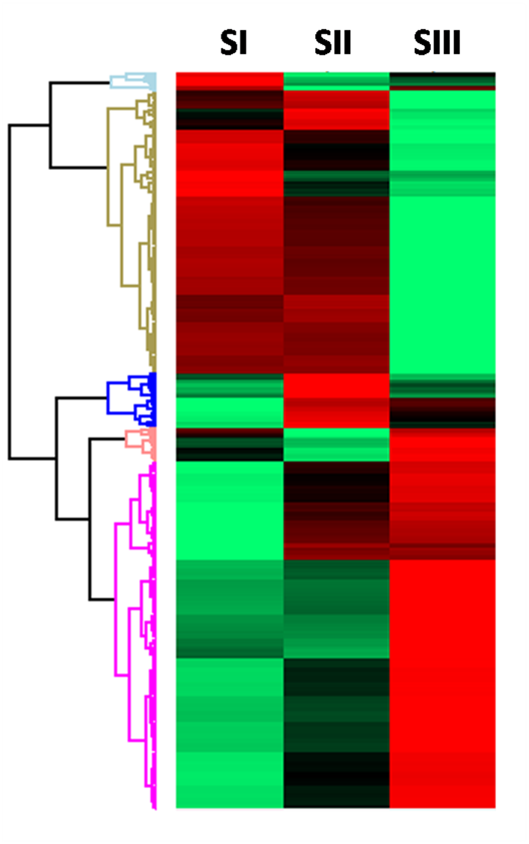

Supplement: FIG S2 [file msystems.00199-22-s0002.tif]

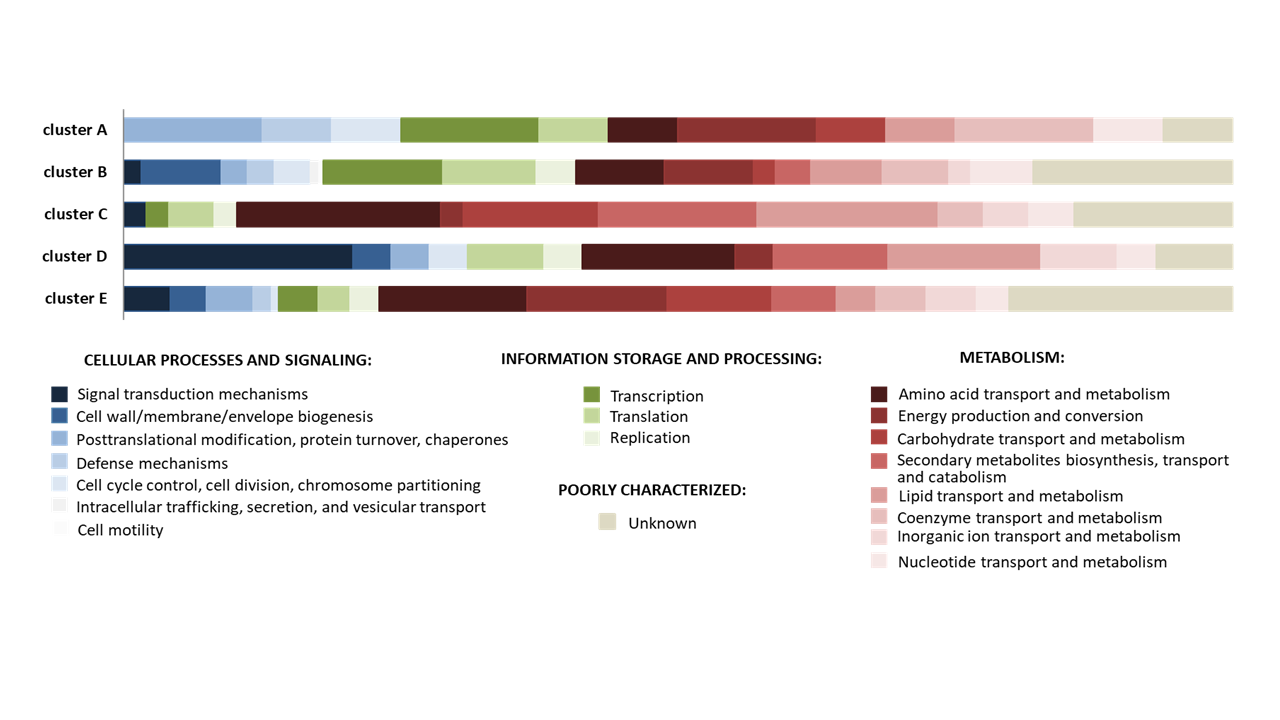

Supplement: FIG S3 [file msystems.00199-22-s0003.tif]
