# Supplementary material for: Phosphoproteome Dynamics of Streptomyces rimosus during Submerged Growth and Antibiotic Production
Source: mSystems. 2022 Sep 12;7(5):e00199-22. doi: 10.1128/msystems.00199-22 (PMC9600765; doi:10.1128/msystems.00199-22)
Supplement: TABLE S1 [file msystems.00199-22-s0005.docx]

| **Enriched category/subcategory/pathway** | **Enrichment factor** | **P-value** | **Benj. Hoch. FDR** |
| --- | --- | --- | --- |
| **Cluster B** | | | |
| RNA polymerases | 5.352 | 0.009383 | 0.17812 |
| Prenyltransferases | 4.7573 | 0.004208 | 0.17812 |
| Peptidoglycan biosynthesis and degradation proteins | 2.8544 | 0.0095 | 0.17812 |
| integral component of membrane | 2.7185 | 0.00406 | 0.097359 |
| integral component of plasma membrane | 2.7185 | 0.00406 | 0.097359 |
| intrinsic component of membrane | 2.7185 | 0.00406 | 0.097359 |
| intrinsic component of plasma membrane | 2.7185 | 0.00406 | 0.097359 |
| Transcription machinery | 2.5949 | 0.005504 | 0.17812 |
| plasma membrane part | 2.4821 | 0.007281 | 0.16194 |
| Amino acid related enzymes | 2.3787 | 0.001152 | 0.086374 |
| Cell wall/membrane/envelope biogenesis | 1.8831 | 0.002049 | 0.023905 |
| Ligases | 1.7608 | 0.004276 | 0.056663 |
| Transcription | 1.6887 | 0.000756 | 0.020706 |
| Transferases | 1.4092 | 0.002275 | 0.056663 |
| INFORMATION STORAGE AND PROCESSING | 1.2545 | 0.007373 | 0.024577 |
| CELLULAR PROCESSES AND SIGNALING | 1.0521 | 0.06474 | 0.11771 |
| POORLY CHARACTERIZED | 1.0413 | 0.076525 | 0.11773 |
| **Cluster C** | | | |
| Lipid transport and metabolism | 2.9843 | 0.000986 | 0.020706 |
| Secondary metabolites biosynthesis, transport and catabolism | 2.8961 | 0.006656 | 0.058244 |
| METABOLISM | 1.583 | 4.04E-05 | 0.000269 |
| **Cluster D** | | | |
| Two-component system | 13.67 | 0.008257 | 0.17812 |
| Signal transduction mechanisms | 3.7664 | 0.003292 | 0.034569 |
| CELLULAR PROCESSES AND SIGNALING | 1.7745 | 0.032228 | 0.064456 |
| METABOLISM | 1.0843 | 0.1347 | 0.1796 |
| **Cluster E** | | | |
| Glutathione metabolism | 4.3442 | 0.000491 | 0.17467 |
| Proteasome | 3.8615 | 0.009015 | 0.17812 |
| Enzymes of 2-oxocarboxylic acid metabolism | 2.3169 | 0.004262 | 0.17812 |
| Carbohydrate transport and metabolism | 1.6549 | 0.000793 | 0.020706 |
| Isomerases | 1.5797 | 0.021075 | 0.15806 |
| Microbial metabolism in diverse environments | 1.4938 | 0.000524 | 0.17467 |
| Energy production and conversion | 1.475 | 0.001739 | 0.022825 |
| Oxidoreductases | 1.361 | 0.005666 | 0.056663 |
| Amino acid transport and metabolism | 1.3556 | 0.00559 | 0.05336 |
| POORLY CHARACTERIZED | 1.243 | 0.014374 | 0.039285 |
| Function unknown | 1.243 | 0.014374 | 0.1161 |
| METABOLISM | 1.2115 | 2.53E-05 | 0.000253 |
