# Supplementary material for: Phosphoproteome Dynamics of Streptomyces rimosus during Submerged Growth and Antibiotic Production
Source: mSystems. 2022 Sep 12;7(5):e00199-22. doi: 10.1128/msystems.00199-22 (PMC9600765; doi:10.1128/msystems.00199-22)
Supplement: TABLE S3 [file msystems.00199-22-s0007.docx]

| **gene ID** | **phosphopeptides alignment** | **predicted function** | **% sim.** | **Source** |
| --- | --- | --- | --- | --- |
| SRIM_024465 | 1AVSLSKGGNV**S**LTK14  \|\|\|\|\|\|\|\|\|\|\|\|\|\|  1AVSL**S**KGGNVSLTK14 | TerD family protein | 90.6 | this study |
| SCO4277 |  | TerD like protein |  | StrepDB |
| SRIM_012680 | 1AVSLSKGGNV**S**LTK14  \|\|\|\|\|\|\|\|\|\|\|\|\|\|  1GVSL**S**KGGNVSLTK14 | TerD family protein | 88.5 | this study |
| SCO2368 |  | TerD like protein |  | StrepDB |
| SRIM_014315 | 471MPPAAAAVPQQFRDDVREE**T**QDDLPVVTERTANGLPQRR509  .\|\|..:: :\|\|:\|.\|\|\|.\|\|.\|\|\|\|\|\|  419-IPATVSL------------EDDVPEVTEW**T**AGGLPQRR444 | sensor histidine kinase | 51 | this study |
| SCO1630 |  | putative membrane protein |  | StrepDB |
| SRIM_010835 | 236QAIEGGQA**S**QGQQAPQAQPGQ--PR258  \|\|\|\|\|\|\| .\|\|:.:.\|:\|..\| \|\|  232QAIEGGQ-GQGEA**SS**QSQQPQDTPR255 | PspA/IM30 family protein | 90 | this study |
| SCO2168 |  | hypothetical protein |  | StrepDB |
| SRIM_001815 | 173LWRQVLAVPLIGTLD**T**TR187  \|\|..::\|\|\|\|\|\|\|\|\|:.\|  175LWDGIVAVPLIGTLD**S**AR192 | STAS domain-containing protein | 41.2 | this study |
| SCO7324 |  | putative regulatory protein |  | StrepDB |
| SRIM_022025 | 1135SQNPQHPQQHLLN**SS**EGR1152  \|......:...:....\|\|  1164SLAGGQGRLAPV**T**EGAGR1181 | PAS domain-containing protein | 54.2 | this study |
| SCO4009 |  | putative bifunctional protein (histidine kinase and regulator) |  | StrepDB |
| SRIM_008500 | 1ERYYDDDYAEGSEPG**ST**GNTPWVTDPR27  \|\|\|\|\|\|\|\|:\|\|.\|.\|.. \|\|\|\|\|\|  1ERYYDDDYSEGPESGDA----WV**T**DPR23 | Cell division protein SepF | 73.5 | this study |
| SCO1749 |  | conserved hypothetical protein |  | StrepDB |
| SRIM_026145 | 148FNASLSTVTATLPAVPS**T**S----166  \|\|.:\|\|...\|.....\|:.:  144FNGALSARMAPSGVSPTDAPSG**S**166 | MarR family transcriptional regulator | 79.1 | this study |
| SCO4961 |  | putative marR-family transcriptional regulator |  | StrepDB |
| SRIM_039385 | 37SPDEGDDEEEHT-DVPDTDV**S**G**T**GRR61  :...\|\|.\|.... \|\|\|\|\|\|.:\|\|\|\|:  57AESAGDAETGPVPDVPD**T**DEAGTGRQ82 | hypothetical protein | 42.4 | this study |
| SCO0793 |  | hypothetical protein |  | StrepDB |
| SRIM_019790 | 64YADLYNRDDDDEDEGN**S**SQQR84  \|\|\|\|\|..\|\|:\|\|\|..:.\|\|\|\|  61YADLYEDDDEDEDGQSP**S**QQR81 | DUF3073 domain-containing protein | 83.8 | this study |
| SCO4088 |  | hypothetical protein |  | StrepDB |
