# Supplementary material for: Phosphoproteome Dynamics of Streptomyces rimosus during Submerged Growth and Antibiotic Production
Source: mSystems. 2022 Sep 12;7(5):e00199-22. doi: 10.1128/msystems.00199-22 (PMC9600765; doi:10.1128/msystems.00199-22)
Supplement: TABLE S4 [file msystems.00199-22-s0008.docx]

| **predicted function** | **SRIM ID** | **% similarity** | **SCO ID** | **predicted function** |
| --- | --- | --- | --- | --- |
| integration host factor | SRIM_015460 | 93.5 | SCO2950 | DNA-binding protein Hu (hs1) |
| citrate synthase | SRIM_014495 | 91.8 | SCO2736 | citrate synthase |
| 30S ribosomal protein S17 | SRIM_018535 | 88.4 | SCO4711 | 30S ribosomal protein S17 |
| NADH-quinone oxidoreductase subunit C | SRIM_018970 | 87.5 | SCO4564 | NuoC, NADH dehydrogenase subunit |
| 30S ribosomal protein S16 | SRIM_029570 | 75.4 | SCO5591 | 30S ribosomal protein S16 |
| histidine phosphatase family protein | SRIM_013710 | 80.5 | SCO2576 | phosphoglycerate mutase |
| FHA domain-containing protein | SRIM_014230 | 82.4 | SCO2669 | hypothetical protein |
| D-alanyl-D-alanine carboxypeptidase | SRIM_017755 | 53.7 | SCO4439 | putative D-alanyl-D-alanine carboxypeptidase |
| FHA domain-containing protein | SRIM_015590 | 45.5 | SCO2975 | hypothetical protein |
| preprotein translocase  subunit YajC | SRIM_006840 | 70.4 | SCO1517 | putative secreted protein |
| Asp23/Gls24 family envelope stress response protein | SRIM_009140 | 79.7 | SCO1836 | putative stress-like protein |
| transcription termination/antitermination protein NusA | SRIM_029960 | 91.5 | SCO5704 | putative transcriptional termination/antitermination factor |
| Serine/threonine-protein kinase | SRIM_017990 | 71 | SCO4423 | serine/threonine-protein kinase AfsK |
| ATP-dependent zinc metalloprotease FtsH | SRIM_023435 | 88.7 | SCO3404 | cell division protein ftsH homolog |
| cell division protein FtsZ | SRIM_010400 | 89.2 | SCO2082 | cell division protein |
| Antitoxin | SRIM_005995 | 44.8 | SCO3108 | conserved hypothetical protein |
| HAMP domain-containing protein | SRIM_029210 | 52.1 | SCO5544 | putative membrane protein |
| sensor histidine kinase | SRIM_027850 | 43.7 | SCO5289 | putative two component sensor kinase |
