# Supplementary material for: Phosphoproteome Dynamics of Streptomyces rimosus during Submerged Growth and Antibiotic Production
Source: mSystems. 2022 Sep 12;7(5):e00199-22. doi: 10.1128/msystems.00199-22 (PMC9600765; doi:10.1128/msystems.00199-22)
Supplement: TABLE S2 [file msystems.00199-22-s0006.docx]

| **SRIM locus tag** | **upregulated protein** | **upregulated phoshorylation site** | **SCO locus tag** | **upregulated protein** | **upregulated phoshorylation site** |
| --- | --- | --- | --- | --- | --- |
| **SRIM_014215** |  |  | SCO1468 | **MIII** |  |
| **SRIM_002990** |  |  | **SCO2666** | **MII** | **MI, MIII** |
| **SRIM_020800** |  | **SI** | **SCO3821** | **MI** | **MI** |
| **SRIM_021000** |  |  | SCO3848 |  |  |
| **SRIM_020795** |  | **SII** | SCO3820 |  |  |
| **SRIM_010555** |  |  | SCO2110 |  |  |
| SRIM_022770 | **SIII** |  | SCO3102 | **MIII** |  |
| SRIM_036860 | **SII, SIII** |  | SCO3893 |  |  |
| **SRIM_021190** |  |  | SCO3344 |  |  |
| **SRIM_017990** |  |  | SCO4488 |  |  |
| **SRIM_007050** | **SIII** | **SI, SII** | SCO4507 |  |  |
| **SRIM_025105** |  | **SII** | SCO3621 | **MIII** |  |
| **SRIM_018190** |  |  | SCO6219 |  |  |
| SRIM_006240 |  |  | SCO4487 |  |  |
| SRIM_027165 |  |  | SCO4377 |  |  |
| SRIM_018185 |  |  | **SCO4423** |  |  |
| SRIM_015585 | **SII** |  | **SCO1551** |  |  |
| **SRIM_015580** |  |  | SCO4779 |  |  |
|  |  |  | SCO4777 |  |  |
|  |  |  | SCO4775 |  |  |
|  |  |  | SCO2450 |  |  |
|  |  |  | SCO4778 | **MII, MIII** |  |
|  |  |  | SCO4776 | **MII, MIII** |  |
|  |  |  | SCO2974 |  |  |
|  |  |  | SCO2973 |  |  |
