# Supplementary material for: Phosphoproteome Dynamics of Streptomyces rimosus during Submerged Growth and Antibiotic Production
Source: mSystems. 2022 Sep 12;7(5):e00199-22. doi: 10.1128/msystems.00199-22 (PMC9600765; doi:10.1128/msystems.00199-22)
Supplement: TABLE S5 [file msystems.00199-22-s0009.docx]

| **Protein** | **Function** | **Gram positive bacteria** | **Gram negative bacteria** | **Archea** |
| --- | --- | --- | --- | --- |
| SRIM_040820 | SDR family oxidoreductase | *Mycobacterium tuberculosis, Mycobacterium bovis*(Actinobacteria)  *Staphylococcus aureus, Bacillus subtilis, Listeria monocytogenes* (Firmicutes) | *Synechocystis sp.* (Cyanobacteria)  *Acinetobacter baumannii, Rhizobium meliloti, Shigella flexneri, Escherichia coli* (Proteobacteria) | *Sulfolobus acidocaldarius, Sulfolobus solfataricus* (Crenarchaeota) |
| SRIM_035205 | NADP-dependent succinic semialdehyde dehydrogenase | *Oenococcus oeni,*  *Bacillus subtilis, Staphylococcus aureus, Clostridium acetobutylicum* (Firmicutes)  *Mycobacterium tuberculosis,*  *Saccharopolyspora erythraea,*  *Mycobacterium bovis* (Actinobacteria) | *Klebsiella pneumoniae,Shigella flexneri, Escherichia coli, Rhodopseudomonas palustris Acinetobacter baumannii (*Proteobacteria)  *Synechocystis sp.* (Cyanobacteria) | *Sulfolobus acidocaldarius,*  *Saccharolobus solfataricus* (Crenarchaeota) |
| SRIM_031215 | 2,3-dihydroxybenzoyl)adenylate synthase | *Mycobacterium tuberculosis,*  *Mycobacterium bovis, Nocardia iowensis,Saccharopolyspora erythraea* (Actinobacteria)  *Bacillus subtilis, Brevibacillus parabrevis,Aneurinibacillus migulanus,Lactococcus lactis, Staphylococcus aureus* (Firmicutes) | *Escherichia coli, Shigella flexneri,*  *Pseudomonas fluorescens, Pseudomonas aeruginosa, Escherichia coli, Acinetobacter baumannii, Enterobacter agglomerans* (Proteobacteria) | *Sulfolobus acidocaldarius, Saccharolobus solfataricus* (Crenarchaeota) |
| SRIM_030485 | vitamin B12-dependent ribonucleotide reductase | *Saccharopolyspora erythraea* (Actinobacteria)  *Bacillus subtilis, Streptococcus pneumoniae* (Firmicutes) | *Rhodopseudomonas palustris,*  *Pseudomonas aeruginosa, Shigella flexneri,* (Proteobacteria)  *Mycoplasma pneumoniae* (Tenericutes) | *Saccharolobus solfataricus,*  *Sulfolobus acidocaldarius* (Crenarchaeota) |
| SRIM_029990 | bifunctional riboflavin kinase/FAD synthetase | *Mycobacterium tuberculosis* (Actinobacteria) | *Pseudomonas fluorescens, Klebsiella pneumoniae, Salmonella newport, Escherichia coli* (Proteobacteria)  *Thermus thermophilus* (Deinococcus-Thermus) |  |
| SRIM_028130 | F0F1 ATP synthase subunit delta | *Mycobacterium bovis, Mycobacterium tuberculosis*  *(Actinobacteria)*  *Bacillus subtilis, Oenococcus oeni (Firmicutes)* | *Synechocystis sp.* (Cyanobacteria) |  |
| SRIM_026575 | 4-hydroxy-3-methylbut-2-enyl diphosphate reductase | *Streptomyces coelicolor* (Actinobacteria) | *Acinetobacter baumannii* (Proteobacteria) *Synechococcus sp.* (Cyanobacteria) |  |
| SRIM_025945 | phospho-sugar mutase | *Mycobacterium bovis , Mycobacterium smegmatis, Saccharopolyspora erythraea*  *Mycobacterium tuberculosis, Streptomyces coelicolor* (Actinobacteria)  *Streptococcus agalactiae, Bacillus subtilis,*  *Staphylococcus aureus, Caldanaerobacter subterraneus subsp. tengcongensis*  *Listeria monocytogenes,Clostridium acetobutylicum, Lactococcus lactis subsp. lactis* (Firmicutes) | *Sinorhizobium meliloti, Xanthomonas campestris, Acinetobacter baumannii,*  *Shigella flexneri, Pseudomonas aeruginosa, Helicobacter pylori, Escherichia coli,*  *Pseudomonas fluorescens, Rhodopseudomonas palustris,*  *Klebsiella pneumoniae, Xanthomonas axonopodis pv. citri, Rhizobium meliloti, Serratia marcescens,*  *Vibrio antiquarius, Citrobacter koseri* (Proteobacteria)  *Mycoplasma pneumoniae* (Tenericutes)  *Synechococcus sp., Arthrospira platensis C1, Synechocystis sp.* (Cyanobacteria)  *Leptospira interrogans serogroup, Icterohaemorrhagiae serovar Lai* (Spirochaetes)  *Thermus thermophilus* (Deinococcus-Thermus) | *Methanohalophilus portucalensis FDF-1, Halobacterium salinarum* (Euryarchaeota) *Sulfolobus acidocaldarius* (Crenarchaeota) |
| SRIM_025275 | succinate--CoA ligase subunit alpha | *Mycobacterium tuberculosis, Saccharopolyspora erythraea, Mycobacterium smegmatis* (Actinobacteria)  *Bacillus subtilis, Staphylococcus aureus* (Firmicutes) | *Acinetobacter baumannii, Shigella flexneri,*  *Escherichia coli, Pseudomonas aeruginosa, Rhodopseudomonas palustris, Sinorhizobium meliloti* (Proteobacteria)  *Thermus thermophilus* (Deinococcus-Thermus) | *Sulfolobus acidocaldarius, Saccharolobus solfataricus* (Crenarchaeota) |
| SRIM_024130 | sulfurtransferase | *Saccharopolyspora erythraea* (Actinobacteria) | *Escherichia coli* (Proteobacteria) | *Sulfolobus acidocaldarius,*  *Saccharolobus solfataricus* (Crenarchaeota) |
| SRIM_023350 | NAD(P)H:quinone oxidoreductase |  | *Shigella flexneri, Escherichia coli* (Proteobacteria) | *Methanohalophilus portucalensis FDF-1* (Euryarchaeota) |
| SRIM_022690 | flavohemoprotein | *Saccharopolyspora erythraea* (Actinobacteria) | *Vibrio vulnificus* (Proteobacteria) |  |
| SRIM_021450 | phosphomethylpyrimidine synthase ThiC | *Bacillus subtilis* (Firmicutes) | *Pseudomonas aeruginosa,*  *Xanthomonas axonopodis pv. citri* (Proteobacteria) |  |
| SRIM_021105 | decarboxylating 6-phosphogluconate dehydrogenase | *Bacillus subtilis, Staphylococcus aureus, Oenococcus oeni* (Firmicutes)  *Mycobacterium bovis* (Actinobacteria) | *Escherichia coli, Shigella flexneri, Klebsiella pneumoniae, Serratia marcescens, Pseudomonas aeruginosa*(Proteobacteria)  *Synechocystis sp.* (Cyanobacteria) |  |
| SRIM_014495 | citrate synthase | *Streptomyces coelicolor,Mycobacterium tuberculosis, Mycobacterium bovis* (Actinobacteria)  *Bacillus subtilis* (Firmicutes) | *Sinorhizobium meliloti, Escherichia coli, Shigella flexneri, Klebsiella pneumoniae* (Proteobacteria) |  |
| SRIM_014025 | aspartate-semialdehyde dehydrogenase | *Bacillus subtilis, Staphylococcus aureus* (Firmicutes)  *Mycobacterium tuberculosis* (Actinobacteria) | *Escherichia coli* (Proteobacteria)  *Synechocystis sp.* (Cyanobacteria) | *Sulfolobus acidocaldarius* (Crenarchaeota) |
| SRIM_009560 | transaldolase | *Mycobacterium bovis, Mycobacterium tuberculosis* (Actinobacteria)  *Thermus thermophilus* (Deinococcus-Thermus)  *Bacillus subtilis* (Firmicutes) | *Escherichia coli, Shigella flexneri,Vibrio alginolyticus* (Proteobacteria)  *Synechocystis sp.* (Cyanobacteria) |  |
| SRIM_007730 | glycerol-3-phosphate dehydrogenase/oxidase | *Saccharopolyspora erythraea* (Actinobacteria)  *Bacillus subtilis* (Firmicutes) | *Escherichia coli, Shigella flexneri, Vibrio alginolyticus*(Proteobacteria) | *Sulfolobus acidocaldarius* (Crenarchaeota) |
| SRIM_001445 | amino acid adenylation domain-containing protein | *Mycobacterium bovis, Mycobacterium tuberculosis, Saccharopolyspora erythraea, Nocardia iowensis* (Actinobacteria)  *Bacillus subtilis, Brevibacillus parabrevis, Aneurinibacillus migulanus, Staphylococcus aureus, Lactococcus lactis* (Firmicutes) | *Synechocystis sp.* (Cyanobacteria) | *Sulfolobus acidocaldarius, Saccharolobus solfataricus* (Crenarchaeota) |
| SRIM_016865 | MogA/MoaB family molybdenum cofactor biosynthesis protein |  | *Campylobacter jejuni, Shigella flexneri, Escherichia coli* (Proteobacteria) | *Sulfolobus acidocaldarius* (Crenarchaeota) |
| SRIM_018390 | type I pantothenate kinase | *Mycobacterium tuberculosis, Mycobacterium bovis, Mycobacterium smegmatis, Saccharopolyspora erythraea* (Actinobacteria)  *Bacillus subtilis, Staphylococcus aureus, Listeria monocytogenes, Streptococcus agalactiae, Lactococcus lactis, Clostridium acetobutylicum, Caldanaerobacter subterraneus* (Firmicutes) | *Synechocystis sp., Synechococcus sp., Arthrospira platensis* (Cyanobacteria)  *Thermus thermophilus* (Deinococcus-Thermus)  *Rhizobium meliloti, Sinorhizobium meliloti, Serratia marcescens, Citrobacter koseri, Escherichia coli, Helicobacter pylori, Acinetobacter baumannii, Pseudomonas aeruginosa, Xanthomonas axonopodis, Serratia marcescens, Pseudomonas fluorescens, Escherichia coli, Klebsiella pneumoniae, Klebsiella pneumoniae, Rhodopseudomonas palustris, Shigella flexneri, Vibrio antiquarius* (Proteobacteria)  *Xanthomonas campestris, Mycoplasma pneumoniae* (Tenericutes)  *Leptospira interrogans* (Spirochaetes) | *Sulfolobus acidocaldarius* (Crenarchaeota)  *Methanohalophilus portucalensis, Halobacterium salinarum* (Euryarchaeota) |
| SRIM_004810 | catalase | *Saccharopolyspora erythraea* (Actinobacteria)  *Bacillus subtilis, Listeria monocytogenes, Staphylococcus aureus* (Firmicutes) | *Acinetobacter baumannii, Xanthomonas axonopodis* (Proteobacteria) |  |
| SRIM_004240 | 3-phosphoshikimate 1-carboxyvinyltransferase | *Mycobacterium bovis* (Actinobacteria) | *Sinorhizobium meliloti, Helicobacter pylori* (Proteobacteria)  *Thermus thermophilus* (Deinococcus-Thermus) | *Saccharolobus solfataricus* (Crenarchaeota) |
| SRIM_040830 | amino acid adenylation domain-containing protein | *Mycobacterium bovis, Mycobacterium tuberculosis, Saccharopolyspora erythraea* (Actinobacteria)  *Brevibacillus parabrevis, Bacillus subtilis, Aneurinibacillus migulanus, Staphylococcus aureus, Lactococcus lactis subsp. lactis* (Firmicutes) | *Pseudomonas fluorescens, Pseudomonas aeruginosa, Escherichia coli, Enterobacter agglomerans, Shigella flexneri, Acinetobacter baumannii* (Proteobacteria)  *Synechocystis sp.* (Cyanobacteria) | *Saccharolobus solfataricus, Sulfolobus acidocaldarius* (Crenarchaeota) |
| SRIM_038670 | enterochelin esterase |  | *Escherichia coli* (Proteobacteria) |  |
| SRIM_033090 | 4-carboxymuconolactone decarboxylase | *Mycobacterium tuberculosis* (Actinobacteria) | *Arthrospira platensis C1* (Cyanobacteria)  *Escherichia coli* (Proteobacteria) |  |
| SRIM_031925 | uroporphyrinogen decarboxylase | *Staphylococcus aureus* (Firmicutes) | *Acinetobacter baumannii* (Proteobacteria)  *Synechocystis sp.* (Cyanobacteria) | *Methanohalophilus portucalensis FDF-1* (Euryarchaeota) |
| SRIM_028145 | F0F1 ATP synthase subunit beta | *Saccharopolyspora erythraea, Mycobacterium tuberculosis* (Actinobacteria)  *Clostridium acetobutylicum, Oenococcus oeni, Listeria monocytogenes, Staphylococcus aureus, Streptococcus agalactiae, Bacillus subtilis* (Firmicutes) | *Shigella flexneri, Escherichia coli,Acinetobacter baumannii, Pseudomonas aeruginosa, Klebsiella pneumoniae subsp. pneumoniae* (Proteobacteria)  *Mycoplasma pneumoniae* (Tenericutes)  *Citrobacter koseri, Synechococcus sp.* (Cyanobacteria)  *Thermus thermophilus* (Deinococcus-Thermus) | *Sulfolobus acidocaldarius, Saccharolobus solfataricus* (Crenarchaeota)  *Methanohalophilus portucalensis FDF-1* (Euryarchaeota) |
| SRIM_023470 | 2-amino-4-hydroxy-6-hydroxymethyldihydropteridine diphosphokinase | *Streptomyces coelicolor* (Actinobacteria) |  | *Sulfolobus acidocaldarius* (Crenarchaeota) |
| SRIM_023100 | dTMP kinase | *Streptomyces coelicolor* (Actinobacteria)  *Staphylococcus aureus* (Firmicutes) |  |  |
| SRIM_018970 | NADH-quinone oxidoreductase subunit C | *Streptomyces coelicolor,Saccharopolyspora erythraea, Mycobacterium smegmatis, Mycobacterium tuberculosis* (Actinobacteria) | *Shigella flexneri, Helicobacter pylori* (Proteobacteria)  *Synechocystis sp.* (Cyanobacteria) | *Sulfolobus acidocaldarius* (Crenarchaeota) |
| SRIM_015325 | 4-hydroxyphenylpyruvate dioxygenase | *Saccharopolyspora erythraea* (Actinobacteria) | *Pseudomonas fluorescens* (Proteobacteria) |  |
| SRIM_014665 | hydroxymethylglutaryl-CoA lyase | *Bacillus subtilis* (Firmicutes)  *Saccharopolyspora erythraea* (Actinobacteria) | *Synechocystis sp.* (Cyanobacteria) | *Sulfolobus acidocaldarius, Saccharolobus solfataricus* (Crenarchaeota)  *Methanohalophilus portucalensis FDF-1* (Euryarchaeota) |
| SRIM_013960 | ribose-5-phosphate isomerase | *Bacillus subtilis* (Firmicutes) | *Helicobacter pylori,Rhizobium meliloti* (Proteobacteria) |  |
| SRIM_009610 | type I glyceraldehyde-3-phosphate dehydrogenase | *Mycobacterium tuberculosis* (Actinobacteria)  *Caldanaerobacter subterraneus subsp. tengcongensis, Bacillus subtilis, Listeria monocytogenes, Listeria welshimeri, Clostridium acetobutylicum, Lactococcus lactis subsp. lactis, Staphylococcus aureus, Streptococcus suis, Oenococcus oeni, Lactobacillus rhamnosus* (Firmicutes) | *Synechocystis sp.* (Cyanobacteria)  *Mycoplasma pneumoniae* (Tenericutes)  *Rhizobium meliloti, Shigella flexneri, Escherichia coli, Acinetobacter baumannii, Pseudomonas aeruginosa* (Proteobacteria) |  |
| SRIM_024090 | transcriptional repressor | *Mycobacterium bovis,Saccharopolyspora erythraea* (Actinobacteria)  *Listeria monocytogenes, Bacillus subtilis* (Firmicutes) | *Escherichia coli* (Proteobacteria)  *Synechocystis sp.* (Cyanobacteria) |  |
| SRIM_012710 | MFS transporter | *Mycobacterium tuberculosis, Mycobacterium bovis* (Actinobacteria) | *Sinorhizobium meliloti* (Proteobacteria) | *Sulfolobus acidocaldarius, Saccharolobus solfataricus* (Crenarchaeota) |
| SRIM_012540 | heavy metal translocating P-type ATPase | *Mycobacterium bovis, Mycobacterium tuberculosis* (Actinobacteria)  *Caldanaerobacter subterraneus* (Firmicutes) | *Pseudomonas aeruginosa, Synechocystis sp.* (Cyanobacteria)  *Helicobacter pylori, Pseudomonas fluorescens, Serratia marcescens* (Proteobacteria) |  |
| SRIM_020390 | alkaline phosphatase | *Bacillus subtilis* (Firmicutes) | *Escherichia coli, Pseudomonas aeruginosa* (Proteobacteria)  *Thermus thermophilus* (Deinococcus-Thermus) |  |
| SRIM_028875 | alpha/beta hydrolase | *Mycobacterium tuberculosis* (Actinobacteria) | *Pseudomonas aeruginosa* (Proteobacteria)  *Synechococcus sp.* (Cyanobacteria) | *Sulfolobus acidocaldarius* (Crenarchaeota) |
| SRIM_013710 | histidine phosphatase family protein | *Mycobacterium tuberculosis, Mycobacterium bovis*(Actinobacteria)  *Lactococcus lactis* (Firmicutes) | *Klebsiella pneumoniae, Shigella flexneri, Escherichia coli, Yersinia pseudotuberculosis* (Proteobacteria) |  |
| SRIM_007705 | HAD family phosphatase | *Lactococcus lactis (Firmicutes)* | *Escherichia coli, Rhizobium meliloti* (Proteobacteria) | *Sulfolobus acidocaldarius* (Crenarchaeota) |
| SRIM_020510 | ATP-binding cassette domain-containing protein | *Streptomyces coelicolor, Mycobacterium tuberculosis, Mycobacterium smegmatis, Saccharopolyspora erythraea, Mycobacterium bovis* (Actinobacteria)  *Listeria monocytogenes, Caldanaerobacter subterraneus, Bacillus subtilis, Lactococcus lactis, Streptococcus pneumoniae, Streptococcus suis, Clostridium acetobutylicum, Staphylococcus aureus, Listeria welshimeri* (Firmicutes) | *Synechococcus sp., Synechocystis sp.* (Cyanobacteria)  *Escherichia coli, Shigella flexneri, Rhizobium meliloti, Pseudomonas fluorescens, Rhodopseudomonas palustris, Acinetobacter baumannii, Sinorhizobium meliloti, Helicobacter pylori* (Proteobacteria)  *Mycoplasma pneumoniae* (Tenericutes) | *Saccharolobus solfataricus, Sulfolobus acidocaldarius* (Crenarchaeota)  *Methanohalophilus portucalensis, Halobacterium salinarum* (Euryarchaeota) |
| SRIM_022485 | class I SAM-dependent methyltransferase |  | *Acinetobacter baumannii* (Proteobacteria) |  |
| SRIM_023135 | non-ribosomal peptide synthetase | *Mycobacterium bovis, Mycobacterium tuberculosis, Saccharopolyspora erythraea* (Actinobacteria)  *Bacillus subtilis, Brevibacillus parabrevis, Aneurinibacillus migulanus, Staphylococcus aureus, Lactococcus lactis*(Firmicutes) | *Synechocystis sp.* (Cyanobacteria*)*  *Pseudomonas aeruginosa, Pseudomonas fluorescens, Escherichia coli, Enterobacter agglomerans, Serratia marcescens, Shigella flexneri, Acinetobacter baumannii* (Proteobacteria) | *Sulfolobus acidocaldarius, Saccharolobus solfataricus* (Crenarchaeota) |
| SRIM_023985 | cytochrome P450 | *Saccharopolyspora erythraea, Streptomyces coelicolor, Mycobacterium tuberculosis, Mycobacterium bovis* (Actinobacteria) |  | *Sulfolobus acidocaldarius* (Crenarchaeota) |
| SRIM_025905 | aldehyde dehydrogenase family protein | *Mycobacterium tuberculosis, Saccharopolyspora erythraea* (Actinobacteria)  *Bacillus subtilis, Staphylococcus aureus, Clostridium acetobutylicum* (Firmicutes) | *Synechocystis sp.* (Cyanobacteria)  *Shigella flexneri, Escherichia coli, Klebsiella pneumoniae, Acinetobacter baumannii, Rhodopseudomonas palustris* (Proteobacteria) | *Sulfolobus acidocaldarius, Saccharolobus solfataricus* (Crenarchaeota) |
| SRIM_027005 | alpha/beta hydrolase | *Mycobacterium bovis* (Actinobacteria) |  | *Saccharolobus solfataricus* (Crenarchaeota) |
| SRIM_031745 | aspartate aminotransferase family protein | *Saccharopolyspora erythraea, Mycobacterium tuberculosis, Mycobacterium bovis* (Actinobacteria)  *Bacillus subtilis, Staphylococcus aureus* (Firmicutes) | *Meiothermus cateniformans, Thermus thermophilus*(Deinococcus-Thermus)  *Synechocystis sp.* (Cyanobacteria)  *Acinetobacter baumannii, Klebsiella pneumoniae, Escherichia coli, Rhizobium meliloti, Shigella flexneri* (Proteobacteria) | *Sulfolobus acidocaldarius* (Crenarchaeota)  *Halobacterium salinarum* (Euryarchaeota) |
| SRIM_032940 | transporter substrate-binding domain-containing protein | *Bacillus subtilis, Streptococcus pneumoniae* (Firmicutes) | *Escherichia coli* (Proteobacteria) |  |
| SRIM_034255 | glucose 1-dehydrogenase | *Mycobacterium tuberculosis, Mycobacterium bovis* (Actinobacteria)  *Staphylococcus aureus, Listeria monocytogenes, Bacillus subtilis* (Firmicutes*)* | *Synechocystis sp., Synechococcus sp.* (Cyanobacteria)  *Rhizobium meliloti, Escherichia coli, Shigella flexneri* (Proteobacteria) | *Saccharolobus solfataricus, Sulfolobus acidocaldarius* (Crenarchaeota) |
| SRIM_036415 | glyceraldehyde-3-phosphate dehydrogenase | *Mycobacterium tuberculosis* (Actinobacteria)  *Bacillus subtilis, Clostridium acetobutylicum, Caldanaerobacter subterraneus, Staphylococcus aureus, Listeria monocytogenes, Listeria welshimeri, Streptococcus suis, Lactococcus lactis, Lactococcus lactis, Lactobacillus rhamnosus, Oenococcus oeni* (Firmicutes) | *Synechocystis sp.* (Cyanobacteria)  *Pseudomonas aeruginosa, Acinetobacter baumannii, Rhizobium meliloti, Shigella flexneri, Escherichia coli* (Proteobacteria)  *Mycoplasma pneumoniae* (Tenericutes) |  |
| SRIM_037420 | SDR family NAD(P)-dependent oxidoreductase | *Mycobacterium bovis, Mycobacterium tuberculosis, Saccharopolyspora erythraea, Streptomyces venezuelae* (Actinobacteria)  *Bacillus subtilis* (Firmicutes) | *Klebsiella pneumoniae, Shigella flexneri, Helicobacter pylori, Escherichia coli, Pseudomonas aeruginosa, Acinetobacter baumannii, Pseudomonas fluorescens* (Proteobacteria) |  |
| SRIM_040360 | cytochrome P450 | *Saccharopolyspora erythraea, Mycobacterium tuberculosis, Streptomyces coelicolor, Mycobacterium bovis*  (Actinobacteria) |  | *Sulfolobus acidocaldarius* (Crenarchaeota) |
| SRIM_040735 | M4 family metallopeptidase | *Bacillus subtilis* (Firmicutes) | *Pseudomonas aeruginosa* (Proteobacteria) |  |
| SRIM_020765 | 2-oxo acid dehydrogenase subunit E2 | *Mycobacterium tuberculosis, Mycobacterium bovis, Saccharopolyspora erythraea*(Actinobacteria)  *Bacillus subtilis, Staphylococcus aureus, Listeria monocytogenes*(Firmicutes) | *Serratia marcescens, Shigella flexneri, Escherichia coli, Rhodopseudomonas palustris, Sinorhizobium meliloti*(Proteobacteria)  *Synechocystis sp.*(Cyanobacteria) |  |
| SRIM_039505 | non-ribosomal peptide synthetase | *Brevibacillus parabrevis, Bacillus subtilis, Aneurinibacillus migulanus, Lactococcus lactis,Staphylococcus aureus*(Firmicutes)  *Mycobacterium bovis, Mycobacterium tuberculosis, Saccharopolyspora erythraea*(Actinobacteria) | *Pseudomonas fluorescens, Pseudomonas aeruginosa, Escherichia coli, Enterobacter agglomerans, Serratia marcescens, Shigella flexneri, Acinetobacter baumannii*(Proteobacteria)  *Synechocystis sp.*(Cyanobacteria) | *Sulfolobus acidocaldarius, Saccharolobus solfataricus*(Crenarchaeota) |
| SRIM_005835 | zinc-binding dehydrogenase | *Mycobacterium bovis, Mycobacterium tuberculosis, Saccharopolyspora erythraea* (Actinobacteria)  *Bacillus subtilis, Oenococcus oeni* (Firmicutes) | *Pseudomonas aeruginosa, Escherichia coli, Pseudomonas fluorescens* (Proteobacteria)  *Thermus thermophilus* (Deinococcus-Thermus) | *Sulfolobus acidocaldarius* (Crenarchaeota) |
| SRIM_003495 | C-terminal binding protein | *Bacillus subtilis* (Firmicutes)  *Saccharopolyspora erythraea, Mycobacterium tuberculosis, Mycobacterium bovis* (Actinobacteria) | *Synechocystis sp.* (Cyanobacteria)  *Shigella flexneri, Helicobacter pylori, Escherichia coli, Campylobacter jejuni* (Proteobacteria) | *Sulfolobus acidocaldarius* (Crenarchaeota) |
| SRIM_028435 | acetate kinase | *Mycobacterium tuberculosis*  *Mycobacterium bovis* (Actinobacteria)  *Clostridium acetobutylicum, Bacillus subtilis, Listeria monocytogenes*(Firmicutes) | *Escherichia coli, Shigella flexneri* (Proteobacteria)  *Mycoplasma pneumoniae* (Tenericutes) |  |
| SRIM_028135 | F0F1 ATP synthase subunit alpha | *Saccharopolyspora erythraea, Mycobacterium tuberculosis* (Actinobacteria)  *Bacillus subtilis, Listeria monocytogenes, Staphylococcus aureus, Streptococcus agalactiae, Oenococcus oeni, Clostridium acetobutylicum* (Firmicutes) | *Synechococcus sp.* (Cyanobacteria)  *Acinetobacter baumannii, Pseudomonas aeruginosa, Citrobacter koseri, Shigella flexneri, Escherichia coli, Klebsiella pneumoniae* (Proteobacteria)  *Mycoplasma pneumoniae* (Tenericutes) *Thermus thermophilus* (Deinococcus-Thermus) | *Sulfolobus acidocaldarius, Saccharolobus solfataricus* (Crenarchaeota)  *Methanohalophilus portucalensis FDF-1* (Euryarchaeota) |
| SRIM_010935 | 2-oxoglutarate dehydrogenase, E2 component, dihydrolipoamide succinyltransferase | *Mycobacterium bovis, Mycobacterium tuberculosis, Saccharopolyspora erythraea*  (Actinobacteria)  *Bacillus subtilis, Staphylococcus aureus, Listeria monocytogenes*(Firmicutes) | *Shigella flexneri, Escherichia coli, Rhodopseudomonas palustris, Serratia marcescens subsp. marcescens, Sinorhizobium meliloti* (Proteobacteria)  *Synechocystis sp.* (Cyanobacteria) |  |
| SRIM_035545 | NAD(P)/FAD-dependent oxidoreductase | *Mycobacterium bovis, Mycobacterium tuberculosis* (Actinobacteria)  *Streptococcus pneumoniae, Bacillus subtilis* (Firmicutes) | *Acinetobacter baumannii, Rhodopseudomonas palustris* (Proteobacteria) | *Sulfolobus acidocaldarius,*  *Saccharolobus solfataricus* (Crenarchaeota) |
| SRIM_004055 | nitrite reductase small subunit NirD | *Mycobacterium bovis, Mycobacterium tuberculosis, Saccharopolyspora erythraea* (Actinobacteria)  *Staphylococcus aureus, Listeria monocytogenes, Listeria welshimeri, Bacillus subtilis, Streptococcus pneumoniae* (Firmicutes) | *Shigella flexneri, Acinetobacter baumannii,*  *Escherichia coli, Rhodopseudomonas palustris* (Proteobacteria)  *Mycoplasma pneumoniae* (Tenericutes) | *Sulfolobus acidocaldarius* (Crenarchaeota) |
| SRIM_034035 | non-ribosomal peptide synthetase | *Mycobacterium tuberculosis, Mycobacterium bovis, Saccharopolyspora erythraea*  (Actinobacteria)  *Bacillus subtilis, Brevibacillus parabrevis*  *Aneurinibacillus migulanus, Staphylococcus aureus, Lactococcus lactis subsp. lactis* (Firmicutes) | *Escherichia coli, Pseudomonas fluorescens, Pseudomonas aeruginosa, Enterobacter agglomerans, Acinetobacter baumannii, Shigella flexneri* (Proteobacteria)  *Synechocystis sp.* (Cyanobacteria) | *Sulfolobus acidocaldarius, Saccharolobus solfataricus* (Crenarchaeota) |
| SRIM_023945 | phosphate ABC transporter permease PstA | *Mycobacterium tuberculosis* (Actinobacteria) | *Escherichia coli, Acinetobacter baumannii* (Proteobacteria) | *Methanohalophilus mahii* (Euryarchaeota) |
| SRIM_016200 | cytosine permease |  |  |  |
| SRIM_020330 | 2,3-diaminopropionate biosynthesis protein SbnB |  |  |  |
| SRIM_018400 | phosphoglucosamine mutase |  |  |  |
| SRIM_021705 | copper resistance protein CopC/CopD |  |  |  |
| SRIM_015305 | VOC family protein |  |  |  |
| SRIM_020395 | TerB family tellurite resistance protein |  |  |  |
| SRIM_005065 | LysE family translocator |  |  |  |
| SRIM_026280 | class IV adenylate cyclase |  |  |  |
| SRIM_034815 | CDP-alcohol phosphatidyltransferase family protein |  |  |  |
| SRIM_004940 | ABC transporter ATP-binding protein | *Mycobacterium bovis, Mycobacterium tuberculosis, Mycobacterium smegmatis,Saccharopolyspora erythraea, Streptomyces coelicolor* (Actinobacteria)  *Bacillus subtilis, Listeria monocytogenes, Listeria welshimeri, Streptococcus pneumoniae, Lactococcus lactis, Streptococcus suis, Clostridium acetobutylicum, Staphylococcus aureus, Caldanaerobacter subterraneus*(Firmicutes) | *Escherichia coli, Rhodopseudomonas palustris, Rhizobium meliloti, Sinorhizobium melilot, Pseudomonas fluorescens, Shigella flexneri, Helicobacter pylori, Acinetobacter baumannii*(Proteobacteria)  *Synechocystis sp., Synechococcus sp.* (Cyanobacteria)  *Mycoplasma pneumoniae* (Tenericutes) | *Saccharolobus solfataricus, Sulfolobus acidocaldarius* (Crenarchaeota)  *Methanohalophilus portucalensis, Halobacterium salinarum* (Euryarchaeota) |
| SRIM_015590 | FHA domain-containing protein | *Streptomyces coelicolor, Saccharopolyspora erythraea, Mycobacterium smegmatis, Mycobacterium tuberculosis, Mycobacterium bovis, Corynebacterium glutamicum*(Actinobacteria)  *Staphylococcus aureus* (Firmicutes) |  |  |
| SRIM_007590 | Sec-independent protein translocase subunit TatA | *Mycobacterium tuberculosis, Mycobacterium bovis (Actinobacteria)* |  |  |
| SRIM_007585 | twin-arginine translocase subunit TatC |  |  | *Sulfolobus acidocaldarius* (Crenarchaeota) |
| SRIM_006840 | preprotein translocase subunit YajC | *Streptomyces coelicolor* (Actinobacteria)  *Bacillus subtilis, Listeria monocytogenes*(Firmicutes) |  |  |
| SRIM_021195 | murein biosynthesis integral membrane protein MurJ | *Mycobacterium tuberculosis, Mycobacterium bovis (Actinobacteria)* |  |  |
| SRIM_030215 | DNA translocase FtsK | *Mycobacterium smegmatis, Mycobacterium bovis, Mycobacterium tuberculosis, Streptomyces coelicolor* (Actinobacteria)  *Clostridium acetobutylicum, Staphylococcus aureus* (Firmicutes) | *Shigella flexneri, Escherichia coli* (Proteobacteria) |  |
| SRIM_027850 | sensor histidine kinase | *Streptomyces coelicolor, Saccharopolyspora erythraea, Mycobacterium tuberculosis, Mycobacterium* bovis (Actinobacteria)  *Listeria monocytogenes, Bacillus subtilis*(Firmicutes) | *Anaeromyxobacter sp., Serratia marcescens, Caulobacter vibrioides, Vibrio campbellii, Acinetobacter baumannii*(Proteobacteria)  *Synechococcus sp., Synechocystis sp.* (Cyanobacteria) | *Methanohalophilus portucalensis* (Euryarchaeota) |
| SRIM_028240 | ATP-binding cassette domain-containing protein | *Streptomyces coelicolor, Mycobacterium bovis, Mycobacterium smegmatis, Mycobacterium tuberculosis, Saccharopolyspora erythraea* (Actinobacteria)  *Listeria monocytogenes, Clostridium acetobutylicum, Streptococcus pneumoniae, Bacillus subtilis, Caldanaerobacter subterraneus subsp. tengcongensis, Staphylococcus aureus, Lactococcus lactis subsp. lactis, Streptococcus suis* (Firmicutes) | *Shigella flexneri, Escherichia coli, Rhizobium meliloti, Acinetobacter baumannii, Pseudomonas fluorescens, Helicobacter pylori* (Proteobacteria)  *Mycoplasma pneumoniae* (Tenericutes)  *Synechocystis sp.* (Cyanobacteria) | *Sulfolobus acidocaldarius,*  *Saccharolobus solfataricus* (Crenarchaeota)  *Methanohalophilus portucalensis FDF-1, Halobacterium salinarum* (Euryarchaeota) |
| SRIM_018645 | DNA-directed RNA polymerase subunit beta | *Mycobacterium tuberculosis* (Actinobacteria)  *Clostridium acetobutylicum,*  *Bacillus subtilis, Staphylococcus aureus,Streptococcus pneumoniae* (Firmicutes) | *Synechocystis sp.* (Cyanobacteria)  *Shigella flexneri, Escherichia coli, Helicobacter pylori* (Proteobacteria) | *Saccharolobus solfataricus, Sulfolobus acidocaldarius* (Crenarchaeota) |
| SRIM_017755 | D-alanyl-D-alanine carboxypeptidase | *Streptomyces coelicolor* (Actinobacteria) | *Escherichia coli* (Proteobacteria) |  |
| SRIM_023630 | ATP-dependent Clp protease ATP-binding subunit | *Saccharopolyspora erythraea, Mycobacterium smegmatis, Mycobacterium tuberculosis, Mycobacterium bovis* (Actinobacteria)  *Bacillus subtilis, Staphylococcus aureus, Clostridium acetobutylicum* (Firmicutes) | *Synechocystis sp.* (Cyanobacteria)  *Shigella flexneri, Escherichia coli, Vibrio vulnificus, Helicobacter pylori, Sinorhizobium meliloti* (Proteobacteria)  *Thermus thermophilus* (Deinococcus-Thermus) | *Saccharolobus solfataricus* (Crenarchaeota) |
| SRIM_027135 | mycoredoxin | *Lactococcus lactis subsp. lactis* (Firmicutes) |  |  |
| SRIM_006810 | peptidylprolyl isomerase | *Streptococcus pneumoniae* (Firmicutes)  *Mycobacterium smegmatis* (Actinobacteria) | *Acinetobacter baumannii, Pseudomonas aeruginosa* (Proteobacteria) |  |
| SRIM_012680 | TerD family protein | *Streptomyces coelicolor* (Actinobacteria)  *Bacillus subtilis* (Firmicutes) |  |  |
| SRIM_024465 | TerD family protein | *Streptomyces coelicolor* (Actinobacteria)  *Bacillus subtilis* (Firmicutes) |  |  |
| SRIM_010835 | PspA/IM30 family protein | *Streptomyces coelicolor* (Actinobacteria)  *Bacillus subtilis, Clostridium acetobutylicum* (Firmicutes) | *Synechocystis sp., Synechococcus sp.*(Cyanobacteria) |  |
| SRIM_026930 | PDZ domain-containing protein | *Mycobacterium bovis, Saccharopolyspora erythraea* (Actinobacteria)  *Listeria monocytogenes*(Firmicutes) | *Escherichia coli, Xanthomonas axonopodis pv. citri* (Proteobacteria) | *Sulfolobus acidocaldarius, Saccharolobus solfataricus* (Crenarchaeota) |
| SRIM_018295 | co-chaperone GroES | *Streptomyces coelicolor, Mycobacterium tuberculosis, Mycobacterium bovis, Saccharopolyspora erythraea, Mycobacterium smegmatis*(Actinobacteria)  *Bacillus subtilis, Listeria monocytogenes, Staphylococcus aureus, Streptococcus agalactiae* (Firmicutes) | *Synechocystis sp.* (Cyanobacteria)  *Acinetobacter baumannii, Shigella flexneri, Escherichia coli, Campylobacter jejuni*(Proteobacteria)  *Mycoplasma pneumoniae* (Tenericutes) *Thermus thermophilus* (Deinococcus-Thermus) |  |
| SRIM_023050 | anti-sigma factor antagonist BldG | *Streptomyces coelicolor, Mycobacterium smegmatis, Mycobacterium tuberculosis* (Actinobacteria)  *Bacillus subtilis, Staphylococcus aureus, Bacillus licheniformis* (Firmicutes) | *Leptospira interrogans* (Spirochaetes)  *Synechocystis sp.* (Cyanobacteria)  *Bordetella bronchiseptica* (Proteobacteria) |  |
| SRIM_022025 | PAS domain-containing protein | *Streptomyces coelicolor*(Actinobacteria)  *Listeria monocytogenes* (Firmicutes) | *Escherichia coli, Sinorhizobium meliloti*(Proteobacteria)  *Synechococcus sp.* (Cyanobacteria) |  |
| SRIM_020800 | protein kinase | *Saccharopolyspora erythraea, Mycobacterium smegmatis, Mycobacterium tuberculosis, Mycobacterium bovis, Streptomyces coelicolor, Corynebacterium glutamicum, Amycolatopsis mediterranei* (Actinobacteria)  *Bacillus subtilis, Streptococcus agalactiae, Listeria monocytogenes, Lactococcus lactis, Staphylococcus aureus, Streptococcus suis, Streptococcus pneumoniae* (Firmicutes) | *Pseudomonas aeruginosa, Vibrio alginolyticus, Serratia marcescens* (Proteobacteria)  *Thermus thermophilus* (Deinococcus-Thermus)  *Synechococcus sp., Synechocystis sp.* (Cyanobacteria)  *Leptospira interrogans* (Spirochaetes) | *Saccharolobus solfataricus, Sulfolobus acidocaldarius* (Crenarchaeota) |
| SRIM_017990 | serine/threonine-protein kinase | *Streptomyces coelicolor, Mycobacterium smegmatis, Mycobacterium tuberculosis, Mycobacterium bovis, Saccharopolyspora erythraea, Corynebacterium glutamicum, Amycolatopsis mediterranei*(Actinobacteria)  *Streptococcus agalactiae, Bacillus subtilis, Streptococcus suis, Streptococcus pneumoniae, Lactococcus lactis, Listeria monocytogenes, Staphylococcus aureus*(Firmicutes) | *Pseudomonas aeruginosa, Vibrio alginolyticus, Serratia marcescens*(Proteobacteria)  *Thermus thermophilus* (Deinococcus-Thermus)  *Synechocystis sp., Synechococcus sp.* (Cyanobacteria)  *Leptospira interrogans* (Spirochaetes) | *Saccharolobus solfataricus, Sulfolobus acidocaldarius*(Crenarchaeota) |
| SRIM_014215 | protein kinase | *Streptomyces coelicolor,Mycobacterium tuberculosis, Mycobacterium bovis, Mycobacterium smegmatis, Corynebacterium glutamicum, Saccharopolyspora erythraea*(Actinobacteria)  *Staphylococcus aureus, Bacillus subtilis, Listeria monocytogenes, Lactococcus lactis, Streptococcus pneumoniae,Streptococcus suis, Streptococcus agalactiae* (Firmicutes) | *Synechocystis sp., Synechococcus sp.* (Cyanobacteria)  *Vibrio alginolyticus, Pseudomonas aeruginosa*(Proteobacteria)  *Thermus thermophilus* (Deinococcus-Thermus) |  |
| SRIM_020980 | FHA domain-containing protein | *Streptomyces coelicolor, Mycobacterium tuberculosis, Mycobacterium bovis, Mycobacterium smegmatis, Corynebacterium glutamicum*(Actinobacteria) |  |  |
| SRIM_014315 | sensor histidine kinase | *Streptomyces coelicolor, Saccharopolyspora erythraea, Mycobacterium tuberculosis, Mycobacterium bovis*(Actinobacteria) | *Escherichia coli, Serratia marcescens* (Proteobacteria) |  |
| SRIM_002105 | EF-hand domain-containing protein | *Streptomyces coelicolor* (Actinobacteria) |  |  |
| SRIM_034905 | tyrosine-protein phosphatase | *Mycobacterium tuberculosis* (Actinobacteria) |  |  |
| SRIM_029210 | HAMP domain-containing protein | *Streptomyces coelicolor, Saccharopolyspora erythraea, Mycobacterium tuberculosis, Mycobacterium bovis* (Actinobacteria) | *Anaeromyxobacter sp.* (Proteobacteria)  *Arthrospira platensis* (Cyanobacteria) | *Methanohalophilus portucalensis*  (Euryarchaeota) |
| SRIM_026815 | hypothetical protein | *Streptomyces coelicolor, Saccharopolyspora erythraea* (Actinobacteria) |  |  |
| SRIM_014620 | hypothetical protein | *Saccharopolyspora erythraea* (Actinobacteria) |  |  |
| SRIM_001300 | STAS domain-containing protein | *Streptomyces coelicolor, Mycobacterium tuberculosis* (Actinobacteria*)*  *Bacillus licheniformis, Bacillus subtilis* (Firmicutes) | *Leptospira interrogans* (Spirochaetes) |  |
| SRIM_001820 | STAS domain-containing protein | *Streptomyces coelicolor, Bacillus subtilis, Moorella thermoacetica, Listeria monocytogenes* (Firmicutes) | *Pseudomonas fluorescens, Xanthomonas campestris*(Proteobacteria)  *Synechococcus sp.* (Cyanobacteria) |  |
| SRIM_025105 | serine/threonine protein kinase | *Mycobacterium smegmatis, Corynebacterium glutamicum, Mycobacterium tuberculosis, Mycobacterium bovis, Saccharopolyspora erythraea, Streptomyces coelicolor, Amycolatopsis mediterranei* (Actinobacteria)  *Bacillus subtilis, Listeria monocytogenes,*  *Streptococcus suis, Streptococcus pneumoniae, Streptococcus agalactiae, Lactococcus lactis subsp. lactis, Staphylococcus aureus* (Firmicutes) | *Pseudomonas aeruginosa, Rhodopseudomonas palustris, Serratia marcescens* (Proteobacteria)  *Synechococcus sp., Vibrio alginolyticus*(Cyanobacteria)  *Leptospira interrogans* (Spirochaetes) *Thermus thermophilus* (Deinococcus-Thermus) | *Sulfolobus acidocaldarius, Saccharolobus solfataricus* (Crenarchaeota) |
| SRIM_021000 | Stk1 family PASTA domain-containing Ser/Thr kinase | *Saccharopolyspora erythraea, Mycobacterium smegmatis, Mycobacterium tuberculosis, Mycobacterium bovis, Corynebacterium glutamicum, Streptomyces coelicolor, Corynebacterium glutamicum,, Amycolatopsis mediterranei*  (Actinobacteria)  *Bacillus subtilis, Lactococcus lactis subsp. lactis, Staphylococcus aureus, Listeria monocytogenes, Streptococcus agalactiae, Streptococcus pneumoniae, Streptococcus suis* (Firmicutes) | *Vibrio alginolyticus, Synechococcus sp.* (Cyanobacteria)  *Pseudomonas aeruginosa, Serratia marcescens, Rhodopseudomonas palustris, Yersinia enterocolitica* (Proteobacteria)  *Leptospira interrogans* (Spirochaetes) *Thermus thermophilus* (Deinococcus-Thermus) | *Sulfolobus acidocaldarius,*  *Saccharolobus solfataricus* (Crenarchaeota) |
| SRIM_020985 | serine/threonine-protein phosphatase | *Mycobacterium tuberculosis, Mycobacterium bovis* (Actinobacteria) | *Pseudomonas aeruginosa, Pseudomonas fluorescens* (Proteobacteria)  *Synechocystis sp.* (Cyanobacteria)  *Mycoplasma pneumoniae* (Tenericutes) |  |
| SRIM_020795 | serine/threonine protein kinase | *Saccharopolyspora erythraea, Mycobacterium smegmatis, Mycobacterium tuberculosis, Mycobacterium bovis, Streptomyces coelicolor, Amycolatopsis mediterranei, Corynebacterium glutamicum* (Actinobacteria)  *Bacillus subtilis, Listeria monocytogenes,*  *Lactococcus lactis subsp. lactis, Streptococcus agalactiae, Staphylococcus aureus, Streptococcus suis, Streptococcus pneumoniae* (Firmicutes) | *Pseudomonas aeruginosa, Vibrio alginolyticus, Serratia marcescens* (Proteobacteria)  *Synechocystis sp.* (Cyanobacteria)  *Leptospira interrogans* (Spirochaetes) *Thermus thermophilus* (Deinococcus-Thermus) | *Sulfolobus acidocaldarius,*  *Saccharolobus solfataricus* (Crenarchaeota) |
| SRIM_018190 | serine/threonine protein kinase | *Streptomyces coelicolor, Mycobacterium tuberculosis, Amycolatopsis mediterranei, Saccharopolyspora erythraea, Mycobacterium smegmatis, Mycobacterium bovis, Corynebacterium glutamicum* (Actinobacteria)  *Listeria monocytogenes, Lactococcus lactis, Streptococcus agalactiae, Bacillus subtilis, Streptococcus suis, Streptococcus pneumoniae, Staphylococcus aureus* (Firmicutes) | *Pseudomonas aeruginosa, Rhodopseudomonas palustris* (Proteobacteria)  *Vibrio alginolyticus, Synechocystis sp.* (Cyanobacteria)  *Serratia marcescens, Leptospira interrogans* (Spirochaetes)  *Thermus thermophilus* (Deinococcus-Thermus) | *Sulfolobus acidocaldarius,*  *Saccharolobus solfataricus* (Crenarchaeota) |
| SRIM_014220 | phosphatase |  | *Synechocystis sp.* (Cyanobacteria) |  |
| SRIM_010555 | Stk1 family PASTA domain-containing Ser/Thr kinase | *Saccharopolyspora erythraea, Mycobacterium tuberculosis, Mycobacterium smegmatis, Corynebacterium glutamicum, Streptomyces coelicolor, Amycolatopsis mediterranei, Mycobacterium bovis* (Actinobacteria)  *Bacillus subtilis, Listeria monocytogenes, Lactococcus lactis, Streptococcus suis, Streptococcus pneumoniae, Streptococcus agalactiae, Staphylococcus aureus* (Firmicutes) | *Pseudomonas aeruginosa, Serratia marcescens* (Proteobacteria)  *Vibrio alginolyticus, Synechocystis sp.* (Cyanobacteria)  *Thermus thermophilus* (Deinococcus-Thermus) | *Sulfolobus acidocaldarius* (Crenarchaeota) |
| SRIM_007050 | protein kinase | *Streptomyces coelicolor, Saccharopolyspora erythraea, Corynebacterium glutamicum, Mycobacterium tuberculosis, Mycobacterium bovis, Mycobacterium smegmatis, Amycolatopsis mediterranei*(Actinobacteria)  *Bacillus subtilis, Streptococcus agalactiae, Staphylococcus aureus, Streptococcus suis,Streptococcus pneumoniae, Lactococcus lactis, Listeria monocytogenes*(Firmicutes) | *Thermus thermophilus* (Deinococcus-Thermus)  *Pseudomonas aeruginosa, Vibrio alginolyticus, Serratia marcescens* (Proteobacteria)  *Synechocystis sp., Synechococcus sp.* (Cyanobacteria) | *Sulfolobus acidocaldarius, Saccharolobus solfataricus* (Crenarchaeota) |
| SRIM_001815 | STAS domain-containing protein | *Streptomyces coelicolor* (Actinobacteria)  *Bacillus subtilis, Listeria monocytogenes, Moorella thermoacetica* (Firmicutes) | *Pseudomonas fluorescens, Xanthomonas campestris*(Proteobacteria) |  |
| SRIM_005235 | FHA domain-containing protein | *Mycobacterium tuberculosis, Mycobacterium bovis, Mycobacterium smegmatis, Corynebacterium glutamicum, Streptomyces coelicolor, Saccharopolyspora erythraea* (Actinobacteria) | *Synechocystis sp., Synechococcus sp.* (Cyanobacteria) |  |
| SRIM_026145 | MarR family transcriptional regulator | *Streptomyces coelicolor* (Actinobacteria)  *Bacillus subtilis* (Firmicutes) |  |  |
| SRIM_021670 | Lrp/AsnC family transcriptional regulator |  | *Shigella flexneri* (Proteobacteria) |  |
| SRIM_031465 | cell division protein SepF | *Streptomyces coelicolor, Mycobacterium tuberculosis, Saccharopolyspora erythraea* (Actinobacteria),  *Streptococcus agalactiae* (Firmicutes) | *Synechocystis sp.* (Cyanobacteria) |  |
| SRIM_023435 | ATP-dependent zinc metalloprotease FtsH | *Streptomyces coelicolor, Saccharopolyspora erythraea, Mycobacterium tuberculosis*(Actinobacteria)  *Lactococcus lactis, Staphylococcus aureus*(Firmicutes) | *Synechocystis sp., Synechococcus sp.* (Cyanobacteria)  *Mycoplasma pneumoniae* (Tenericutes)  *Sinorhizobium meliloti, Shigella flexneri, Escherichia coli, Pseudomonas aeruginosa* (Proteobacteria) | *Saccharolobus solfataricus, Sulfolobus acidocaldarius* (Crenarchaeota)  *Haloferax volcanii* (Euryarchaeota) |
| SRIM_010400 | cell division protein FtsZ | *Streptomyces coelicolor, Saccharopolyspora erythraea, Mycobacterium tuberculosis, Mycobacterium smegmatis, Staphylococcus aureus* (Actinobacteria)  *Bacillus subtilis, Streptococcus agalactiae* (Firmicutes) | *Shigella flexneri, Escherichia coli* (Proteobacteria)  *Deinococcus radiodurans* (Deinococcus-Thermus) |  |
| SRIM_010385 | cell division protein SepF | *Streptomyces coelicolor, Saccharopolyspora erythraea, Mycobacterium tuberculosis* (Actinobacteria)  *Staphylococcus aureus, Streptococcus agalactiae* (Firmicutes) | *Synechocystis sp.* (Cyanobacteria) |  |
| SRIM_010375 | DivIVA domain-containing protein | *Streptomyces coelicolor, Saccharopolyspora erythraea, Mycobacterium tuberculosis, Mycobacterium bovis, Mycobacterium smegmatis*(Actinobacteria)  *Listeria monocytogenes, Bacillus subtilis, Streptococcus agalactiae, Streptococcus pneumoniae, Streptococcus suis, Staphylococcus aureus* (Firmicutes) |  |  |
| SRIM_020990 | FtsW/RodA/SpoVE family cell cycle protein | *Mycobacterium tuberculosis*  *Mycobacterium bovis* (Actinobacteria)  *Caldanaerobacter subterraneus subsp. tengcongensis* (Firmicutes) |  |  |
| SRIM_018315 | FtsW/RodA/SpoVE family cell cycle protein | *Mycobacterium tuberculosis, Mycobacterium bovis* (Actinobacteria)  *Caldanaerobacter subterraneus (*Firmicutes) |  |  |
| SRIM_002990 | protein kinase | *Mycobacterium tuberculosis, Mycobacterium bovis, Corynebacterium glutamicum, Streptomyces coelicolor, Mycobacterium smegmatis, Saccharopolyspora erythraea, Mycobacterium tuberculosis, Amycolatopsis mediterranei* (Actinobacteria)  *Lactococcus lactis, Streptococcus agalactiae, Streptococcus suis, Listeria monocytogenes, Streptococcus pneumoniae, Staphylococcus aureus, Bacillus subtilis* (Firmicutes) | *Synechocystis sp., Synechococcus sp.* (Cyanobacteria)  *Vibrio alginolyticus, Pseudomonas aeruginosa* (Proteobacteria)  *Thermus thermophilus* (Deinococcus-Thermus) |  |
| SRIM_003945 | ABC transporter ATP-binding protein | *Mycobacterium bovis, Mycobacterium tuberculosis, Streptomyces coelicolor, Mycobacterium smegmatis, Saccharopolyspora erythraea* (Actinobacteria)  *Listeria monocytogenes, Streptococcus pneumoniae, Lactococcus lactis, Bacillus subtilis, Streptococcus suis, Staphylococcus aureus, Caldanaerobacter subterraneus, Clostridium acetobutylicum* (Firmicutes) | *Synechocystis sp., Synechococcus sp.* (Cyanobacteria)  *Escherichia coli, Rhodopseudomonas palustris, Sinorhizobium meliloti, Pseudomonas fluorescens, Shigella flexneri, Rhizobium meliloti, Helicobacter pylori, Acinetobacter baumannii*(Proteobacteria)  *Mycoplasma pneumoniae* (Tenericutes) | *Saccharolobus solfataricus, Sulfolobus acidocaldarius* (Crenarchaeota)  *Methanohalophilus portucalensis, Halobacterium salinarum* (Euryarchaeota) |
| SRIM_004755 | HAMP domain-containing histidine kinase | *Mycobacterium tuberculosis, Mycobacterium bovis* (Actinobacteria)  *Listeria monocytogenes, Bacillus subtilis, Staphylococcus aureus, Staphylococcus aureus* (Firmicutes) | *Synechococcus sp., Synechocystis sp.* (Cyanobacteria)  *Serratia marcescens, Xanthomonas campestris, Pseudomonas aeruginosa, Pseudomonas aeruginosa, Vibrio campbellii, Escherichia coli, Caulobacter vibrioides, Rhodopseudomonas palustrus, Salmonella typhimurium, Bordetella pertussis* (Proteobacteria)  *Leptospira interrogans* (Spirochaetes) | *Methanohalophilus portucalensis* (Euryarchaeota) |
| SRIM_007275 | response regulator transcription factor | *Mycobacterium tuberculosis, Mycobacterium bovis, Streptomyces coelicolor, Streptomyces reticuli, Saccharopolyspora erythraea* (Actinobacteria)  *Bacillus subtilis, Caldanaerobacter subterraneus, Staphylococcus aureus, Geobacillus stearothermophilus, Clostridium acetobutylicum, Streptococcus pyogenes* (Firmicutes) | *Synechococcus sp., Synechocystis sp., Microchaete diplosiphon, Arthrospira platensis* (Cyanobacteria)  *Escherichia coli, Shigella flexneri, Pseudomonas aeruginosa, Vibrio vulnificus, Rhizobium meliloti, Acinetobacter baumannii, Vibrio campbellii, Azoarcus sp., Salmonella typhimurium, Brucella abortus, Xanthomonas campestris, Rhodopseudomonas palustris, Serratia marcescens, Caulobacter vibrioides* (Proteobacteria)  *Leptospira interrogans*(Spirochaetes)  *Thermotoga maritima* (Thermotogae)  *Thermus thermophilus* (Deinococcus-Thermus) | *Halobacterium salinarum, Methanosarcina mazei* (Euryarchaeota) |
| SRIM_014230 | FHA domain-containing protein | *Streptomyces coelicolor, Mycobacterium tuberculosis, Mycobacterium bovis, Mycobacterium smegmatis* (Actinobacteria) |  |  |
| SRIM_020400 | TerD family protein | *Streptomyces coelicolor* (Actinobacteria)  *Bacillus subtilis* (Firmicutes) |  |  |
| SRIM_021150 | AAA family ATPase | *Mycobacterium tuberculosis, Mycobacterium bovis* (Actinobacteria) |  |  |
| SRIM_021190 | serine/threonine protein kinase | *Corynebacterium glutamicum, Mycobacterium smegmatis, Streptomyces coelicolor, Mycobacterium tuberculosis, Mycobacterium bovis, Saccharopolyspora erythraea, Amycolatopsis mediterranei* (Actinobacteria)  *Bacillus subtilis, Listeria monocytogenes, Streptococcus agalactiae, Lactococcus lactis subsp. lactis, Streptococcus suis, Streptococcus pneumoniae, Staphylococcus aureus* (Firmicutes) |  |  |
| SRIM_028105 | undecaprenyl/decaprenyl-phosphate alpha-N-acetylglucosaminyl 1-phosphate transferase | *Staphylococcus aureus* (Firmicutes) | *Helicobacter pylori, Escherichia coli, Acinetobacter baumannii* (Proteobacteria) |  |
| SRIM_028665 | ABC transporter ATP-binding protein | *Mycobacterium bovis, Mycobacterium tuberculosis, Streptomyces coelicolor, Mycobacterium smegmatis, Saccharopolyspora erythraea* (Actinobacteria)  *Bacillus subtilis, Lactococcus lactis, Streptococcus pneumoniae, Listeria monocytogenes, Clostridium acetobutylicum, Streptococcus suis, Caldanaerobacter subterraneus, Staphylococcus aureus, Listeria welshimeri*(Firmicutes) | *Synechocystis sp., Synechococcus sp. (*Cyanobacteria)  *Escherichia coli, Rhodopseudomonas palustris, Pseudomonas fluorescens, Sinorhizobium meliloti, Helicobacter pylori, Rhizobium meliloti, Shigella flexneri, Acinetobacter baumannii* (Proteobacteria)  *Mycoplasma pneumoniae* (Tenericutes) | *Saccharolobus solfataricus, Sulfolobus acidocaldarius* (Crenarchaeota)  *Halobacterium salinarum, Methanohalophilus portucalensis (Euryarchaeota)* |
| SRIM_030205 | HAMP domain-containing protein | *Mycobacterium tuberculosis, Mycobacterium bovis, Saccharopolyspora erythraea* (Actinobacteria)  *Bacillus subtilis, Listeria monocytogenes, Staphylococcus aureus, Streptococcus pyogenes, Staphylococcus aureus, Geobacillus stearothermophilus, Clostridium acetobutylicum* (Firmicutes) | *Thermus thermophilus* (Deinococcus-Thermus)  *Synechocystis sp., Synechococcus sp., Arthrospira platensis, Microchaete diplosiphon* (Cyanobacteria)  *Leptospira interrogans* (Spirochaetes)  *Vibrio vulnificus, Vibrio campbellii, Xanthomonas campestris, Escherichia coli, Bordetella pertussis, Pseudomonas aeruginosa, Rhodopseudomonas palustris, Caulobacter vibrioides, Brucella abortus, Acinetobacter baumannii, Klebsiella pneumoniae, Shigella flexneri, Salmonella typhimurium, Pseudomonas aeruginosa, Anaeromyxobacter sp., Serratia marcescens, Xanthomonas axonopodis, Salmonella choleraesuis, Klebsiella pneumoniae, Rhodobacter capsulatus* (Proteobacteria)  *Thermotoga maritima* (Thermotogae) | *Methanohalophilus portucalensis, Methanosarcina mazei, Halobacterium salinarum* (Euryarchaeota) |
| SRIM_008500 | cell division protein SepF | *Saccharopolyspora erythraea, Mycobacterium tuberculosis, Streptomyces coelicolor* (Actinobacteria)  *Staphylococcus aureus, Streptococcus agalactiae* (Firmicutes) | *Synechocystis sp.* (Cyanobacteria*)* |  |
| SRIM_015580 | serine/threonine protein kinase | *Mycobacterium bovis, Mycobacterium tuberculosis, Mycobacterium smegmatis, Corynebacterium glutamicum, Saccharopolyspora erythraea, Streptomyces coelicolor, Amycolatopsis mediterranei*(Actinobacteria)  *Streptococcus agalactiae, Bacillus subtilis Lactococcus lactis, Listeria monocytogenes, Streptococcus suis, Streptococcus pneumoniae, Staphylococcus aureus* (Firmicutes) | *Pseudomonas aeruginosa, Vibrio alginolyticus*(Proteobacteria)  *Thermus thermophilus* (Deinococcus-Thermus)  *Synechococcus sp., Synechocystis sp.*(Cyanobacteria) |  |
| SRIM_028210 | ATP-binding protein |  |  |  |
| SRIM_041150 | hypothetical protein |  |  |  |
| SRIM_010620 | sensor histidine kinase |  |  |  |
| SRIM_014925 | peptidase |  |  |  |
| SRIM_021470 | serine/threonine protein phosphatase |  |  |  |
| SRIM_014225 | VWA domain-containing protein |  |  |  |
| SRIM_015925 | LCP family protein | *Streptomyces coelicolor, Mycobacterium tuberculosis, Mycobacterium bovis* (Actinobacteria)  *Staphylococcus aureus* (Firmicutes) |  |  |
| SRIM_007210 | arginine repressor | *Bacillus subtilis* (Firmicutes) |  |  |
| SRIM_019050 | cold-shock protein | *Saccharopolyspora erythraea, Mycobacterium tuberculosis, Mycobacterium bovis* (Actinobacteria) | *Escherichia coli, Rhodopseudomonas palustris* (Proteobacteria) |  |
| SRIM_007455 | 50S ribosomal protein L20 |  | *Escherichia coli, Shigella flexneri (Proteobacteria)* |  |
| SRIM_030245 | DNA starvation/stationary phase protection protein | *Listeria monocytogenes, Lactococcus lactis subsp. lactis, Bacillus subtilis (Firmicutes)* | *Pseudomonas fluorescens, Shigella flexneri,*  *Escherichia coli* (Proteobacteria)  *Synechocystis sp.* (Cyanobacteria) |  |
| SRIM_018615 | elongation factor Tu | *Saccharopolyspora erythraea, Mycobacterium tuberculosis, Mycobacterium bovis* (Actinobacteria)  *Listeria monocytogenes, Bacillus subtilis, Staphylococcus aureus, Streptococcus pneumoniae, Streptococcus suis, Streptococcus agalactiae, Clostridium acetobutylicum, Lactobacillus rhamnosus, Lactococcus lactis subsp. lactis, Bacillus velezensis, Listeria monocytogenes* (Firmicutes) | *Rhodopseudomonas palustris, Serratia marcescens, Escherichia coli, Shigella flexneri, Klebsiella pneumoniae, Sinorhizobium meliloti, Pseudomonas aeruginosa, Rhizobium meliloti, Campylobacter jejuni subsp. jejuni, Acinetobacter baumannii* (Proteobacteria)  *Synechocystis sp.* (Cyanobacteria)  *Mycoplasma pneumoniae, Mycoplasma genitalium* (Tenericutes)  *Thermus thermophilus* (Deinococcus-Thermus) | *Sulfolobus acidocaldarius* (Crenarchaeota)  *Methanohalophilus portucalensis FDF-1, Halobacterium salinarum* (Euryarchaeota) |
| SRIM_018535 | 30S ribosomal protein S17 | *Streptomyces coelicolor, Mycobacterium tuberculosis* (Actinobacteria) | *Klebsiella pneumoniae, Shigella flexneri, Escherichia coli, Citrobacter koseri* (Proteobacteria) |  |
| SRIM_028075 | 50S ribosomal protein L31 | *Mycobacterium tuberculosis* (Actinobacteria)  *Bacillus subtilis, Staphylococcus aureus,*  *Listeria monocytogenes*(Firmicutes) | *Pseudomonas aeruginosa, Escherichia coli, Klebsiella pneumoniae* (Proteobacteria)  *Synechocystis sp.* (Cyanobacteria) |  |
| SRIM_015460 | integration host factor | *Streptomyces coelicolor* (Actinobacteria)  *Staphylococcus aureus, Bacillus subtilis, Listeria monocytogenes, Listeria welshimeri* (Firmicutes) | *Synechococcus sp.* (Cyanobacteria) *Acinetobacter baumannii, Escherichia coli, Rhizobium meliloti, Sinorhizobium meliloti, Rhodopseudomonas palustris, Xanthomonas axonopodis* (Proteobacteria) |  |
| SRIM_029570 | 30S ribosomal protein S16 | *Streptomyces coelicolor, Mycobacterium smegmatis, Saccharopolyspora erythraea, Mycobacterium tuberculosis, Mycobacterium bovis* (Actinobacteria)  *Bacillus subtilis* (Firmicutes) | *Shigella flexneri, Escherichia coli* (Proteobacteria) |  |
| SRIM_029330 | HU family DNA-binding protein | *Mycobacterium bovis, Mycobacterium tuberculosis, Streptomyces coelicolor* (Actinobacteria)  *Bacillus subtilis, Staphylococcus aureus,*  *Listeria monocytogenes, Listeria welshimeri serovar 6b* (Firmicutes) | *Synechocystis sp.* (Cyanobacteria) *Acinetobacter baumannii, Escherichia coli, Rhizobium meliloti, Sinorhizobium meliloti, Escherichia coli, Rhodopseudomonas palustris* (Proteobacteria) |  |
| SRIM_029980 | 30S ribosome-binding factor RbfA | *Mycobacterium bovis* (Actinobacteria) | *Escherichia coli* (Proteobacteria) |  |
| SRIM_029960 | transcription termination/antitermination protein NusA | *Streptomyces coelicolor, Mycobacterium bovis* (Actinobacteria)  *Bacillus subtilis, Staphylococcus aureus*(Firmicutes) | *Shigella flexneri*(Proteobacteria)  *Mycoplasma pneumoniae*(Tenericutes) |  |
| SRIM_024360 | CarD family transcriptional regulator | *Streptomyces coelicolor, Mycobacterium tuberculosis, Mycobacterium bovis*(Actinobacteria) |  |  |
| SRIM_003375 | aminoglycoside phosphotransferase family protein |  | *Rhizobium meliloti* (Proteobacteria)  *Thermus thermophilus* (Deinococcus-Thermus) |  |
| SRIM_021055 | DNA-binding protein | *Streptomyces coelicolor* (Actinobacteria) |  |  |
| SRIM_019760 | ATP-dependent RNA helicase HrpA |  | *Pseudomonas fluorescens, Acinetobacter baumannii* (Proteobacteria) |  |
| SRIM_039930 | TetR/AcrR family transcriptional regulator | *Bacillus subtilis* (Firmicutes)  *Streptomyces coelicolor, Mycobacterium tuberculosis* (Actinobacteria) | *Acinetobacter baumannii* (Proteobacteria) |  |
| SRIM_032170 | 3'-5' exonuclease |  | *Mycoplasma pneumoniae* (Tenericutes) |  |
| SRIM_002125 | helix-turn-helix transcriptional regulator | *Staphylococcus aureus* (Firmicutes) | *Pseudomonas fluorescens, Pseudomonas aeruginosa, Escherichia coli, Enterobacter agglomerans, Serratia marcescens, Shigella flexneri, Acinetobacter baumannii, Shigella flexneri* (Proteobacteria) |  |
| SRIM_006655 | integration host factor | *Mycobacterium tuberculosis, Mycobacterium bovis* (Actinobacteria) |  |  |
| SRIM_015385 | transcriptional regulator | *Streptomyces coelicolor* (Actinobacteria) |  |  |
| SRIM_021285 | replicative DNA helicase | *Staphylococcus aureus* (Firmicutes) | *Synechocystis sp., Synechococcus sp.* (Cyanobacteria)  *Shigella flexneri, Escherichia coli (Proteobacteria)* |  |
| SRIM_021640 | FadR family transcriptional regulator | *Streptomyces coelicolor* (Actinobacteria)  *Bacillus subtilis, Listeria monocytogenes* (Firmicutes) | *Sinorhizobium meliloti, Shigella flexneri* (Proteobacteria) |  |
| SRIM_029730 | Lrp/AsnC family transcriptional regulator |  | *Shigella flexneri, Acinetobacter baumannii, Escherichia coli, Pseudomonas aeruginosa* (Proteobacteria) |  |
| SRIM_008415 | pyridoxamine 5'-phosphate oxidase family protein |  |  |  |
| SRIM_021080 | DUF3566 domain-containing protein | *Saccharopolyspora erythraea, Mycobacterium smegmatis, Mycobacterium tuberculosis, Mycobacterium bovis* (Actinobacteria) |  |  |
| SRIM_019790 | DUF3073 domain-containing protein | *Streptomyces coelicolor,Mycobacterium tuberculosis, Mycobacterium bovis* (Actinobacteria) |  |  |
| SRIM_018745 | YajQ family cyclic di-GMP-binding protein | *Mycobacterium tuberculosis, Mycobacterium bovis* (Actinobacteria) | *Escherichia coli* (Proteobacteria)  *Synechococcus sp.*(Cyanobacteria) |  |
| SRIM_016410 | hypothetical protein | *Streptomyces coelicolor* (Actinobacteria) |  |  |
| SRIM_013125 | general stress protein | *Bacillus subtilis* (Firmicutes) |  |  |
| SRIM_011000 | DUF4191 domain-containing protein | *Mycobacterium tuberculosis, Mycobacterium bovis* (Actinobacteria) |  |  |
| SRIM_008900 | YbhB/YbcL family Raf kinase inhibitor-like protein | *Mycobacterium tuberculosis* (Actinobacteria) |  |  |
| SRIM_039385 | hypothetical protein | *Streptomyces coelicolor* (Actinobacteria) |  |  |
| SRIM_024125 | DUF1416 domain-containing protein | *Streptomyces coelicolor,Saccharopolyspora erythraea, Mycobacterium tuberculosis* (Actinobacteria) |  |  |
| SRIM_039670 | nitroreductase family deazaflavin-dependent oxidoreductase | *Mycobacterium tuberculosis, Mycobacterium bovis* (Actinobacteria) |  |  |
| SRIM_019835 | Lsr2 family protein | *Streptomyces coelicolor,Mycobacterium tuberculosis, Mycobacterium bovis* (Actinobacteria) |  |  |
| SRIM_009140 | Asp23/Gls24 family envelope stress response protein | *Streptomyces coelicolor* (Actinobacteria)  *Staphylococcus aureus, Lactococcus lactis, Streptococcus agalactiae* (Firmicutes) |  |  |
| SRIM_013715 | ribosome silencing factor |  | *Pseudomonas aeruginosa* (Proteobacteria) |  |
| SRIM_022055 | NAD(P)H-dependent oxidoreductase | *Bacillus subtilis* (Firmicutes) | *Shigella flexneri* (Proteobacteria) |  |
| SRIM_006750 | endolytic transglycosylase MltG | *Streptococcus suis, Streptococcus agalactiae, Lactococcus lactis*(Firmicutes) |  |  |
| SRIM_028925 | SDR family oxidoreductase | *Mycobacterium tuberculosis, Mycobacterium bovis, Saccharopolyspora erythraea* (Actinobacteria)  *Bacillus subtilis, Staphylococcus aureus* (Firmicutes) | *Synechocystis sp.* (Cyanobacteria)  *Shigella flexneri, Escherichia coli, Acinetobacter baumannii, Rhizobium meliloti* (Proteobacteria) | *Sulfolobus acidocaldarius, Saccharolobus solfataricus* (Crenarchaeota) |
| SRIM_031615 | hypothetical protein | *Mycobacterium tuberculosis* (Actinobacteria) |  |  |
| SRIM_005995 | antitoxin | *Mycobacterium bovis, Mycobacterium tuberculosis, Streptomyces coelicolor* (Actinobacteria) |  |  |
| SRIM_030020 | WXG100 family type VII secretion target | *Mycobacterium tuberculosis* (Actinobacteria) |  |  |
| SRIM_032155 | hypothetical protein |  |  |  |
| SRIM_014460 | MarR family transcriptional regulator |  |  |  |
| SRIM_029625 | DUF2469 domain-containing protein |  |  |  |
| SRIM_028800 | enhanced serine sensitivity protein SseB |  |  |  |
| SRIM_027495 | hypothetical protein |  |  |  |
| SRIM_024700 | DUF3027 domain-containing protein |  |  |  |
| SRIM_020105 | lYbjN domain-containing protein |  |  |  |
| SRIM_020050 | DUF3151 domain-containing protein |  |  |  |
| SRIM_016615 | hypothetical protein |  |  |  |
| SRIM_016345 | hypothetical protein |  |  |  |
| SRIM_007840 | DUF3090 domain-containing protein |  |  |  |
| SRIM_004435 | hypothetical protein |  |  |  |
| SRIM_041300 | hypothetical protein |  |  |  |
| SRIM_037200 | DUF317 domain-containing protein |  |  |  |
| SRIM_032285 | DUF2795 domain-containing protein |  |  |  |
| SRIM_025665 | hypothetical protein |  |  |  |
| SRIM_024965 | hypothetical protein |  |  |  |
| SRIM_023855 | hypothetical protein |  |  |  |
| SRIM_022155 | hypothetical protein |  |  |  |
| SRIM_021045 | DUF5324 family protein |  |  |  |
| SRIM_010030 | hypothetical protein |  |  |  |
| SRIM_004815 | hypothetical protein |  |  |  |
| SRIM_014615 | hypothetical protein |  |  |  |
| SRIM_026585 | hypothetical protein |  |  |  |
| SRIM_004440 | DUF2795 domain-containing protein |  |  |  |
| SRIM_023405 | threonine/serine exporter family protein |  |  |  |
| SRIM_003100 | hypothetical protein |  |  |  |
| SRIM_006765 | hypothetical protein |  |  |  |
| SRIM_008570 | gas vesicle protein |  |  |  |
| SRIM_019160 | hypothetical protein |  |  |  |
| SRIM_021335 | hypothetical protein |  |  |  |
| SRIM_021445 | YibE/F family protein |  |  |  |
| SRIM_025340 | DUF2690 domain-containing protein |  |  |  |
| SRIM_025805 | ABC transporter permease |  |  |  |
| SRIM_030035 | hypothetical protein |  |  |  |
| SRIM_037305 | hypothetical protein |  |  |  |
| SRIM_037960 | caspase family protein |  |  |  |
| SRIM_039000 | HAD family hydrolase |  |  |  |
| SRIM_000380 | hypothetical protein |  |  |  |

| **Metabolism** | | | |
| --- | --- | --- | --- |
| Gram + | Firmicutes | | SRIM_040820, SRIM_035205, SRIM_031215, SRIM_030485, SRIM_028130, SRIM_025945, SRIM_025275, SRIM_021450, SRIM_021105, SRIM_014495, SRIM_014025, SRIM_009560, SRIM_007730, SRIM_004810, SRIM_040830, SRIM_031925, SRIM_028145, SRIM_023100, SRIM_014665, SRIM_013960, SRIM_009610, SRIM_005835, SRIM_003495, SRIM_028435, SRIM_028135, SRIM_010935, SRIM_035545, SRIM_034035, SRIM_024090, SRIM_012540, SRIM_020390, SRIM_001445, SRIM_007705, SRIM_018390, SRIM_031745, SRIM_020765, SRIM_039505, SRIM_032940, SRIM_020510, SRIM_023135, SRIM_025905, SRIM_034255, SRIM_036415, SRIM_037420, SRIM_040735 |
|  | Actinobacteria | | SRIM_040820, SRIM_035205, SRIM_031215, SRIM_030485, SRIM_029990, SRIM_028130, SRIM_026575, SRIM_025945, SRIM_025275, SRIM_024130, SRIM_022690, SRIM_021105, SRIM_014495, SRIM_014025, SRIM_009560, SRIM_007730, SRIM_004240, SRIM_040830, SRIM_033090, SRIM_028145, SRIM_023470, SRIM_023100, SRIM_018970, SRIM_015325, SRIM_014665, SRIM_009610, SRIM_005835, SRIM_003495, SRIM_028435, SRIM_028135, SRIM_010935, SRIM_035545, SRIM_034035, SRIM_023945, SRIM_024090, SRIM_012540, SRIM_012540, SRIM_028875, SRIM_013710, SRIM_001445, SRIM_018390, SRIM_020510, SRIM_023135, SRIM_020765, SRIM_039505, SRIM_023985, SRIM_025905, SRIM_027005, SRIM_031745, SRIM_034255, SRIM_036415, SRIM_037420, SRIM_040360 |
| Gram - | Proteobacteria | | SRIM_040820, SRIM_035205, SRIM_031215, SRIM_030485, SRIM_029990, SRIM_026575, SRIM_025945, SRIM_025275, SRIM_024130, SRIM_023350, SRIM_022690, SRIM_021450, SRIM_021105, SRIM_014495, SRIM_014025, SRIM_009560, SRIM_007730, SRIM_004810, SRIM_004240, SRIM_040830, SRIM_038670, SRIM_033090, SRIM_028145, SRIM_018970, SRIM_015325, SRIM_013960, SRIM_009610, SRIM_005835, SRIM_003495, SRIM_028435, SRIM_028135, SRIM_010935, SRIM_035545, SRIM_004055, SRIM_034035, SRIM_023945, SRIM_024090, SRIM_012540, SRIM_012540, SRIM_020390, SRIM_028875, SRIM_007705, SRIM_016865, SRIM_018390, SRIM_022485, SRIM_031745, SRIM_020765, SRIM_039505, SRIM_032940, SRIM_020510, SRIM_023135, SRIM_025905, SRIM_034255, SRIM_037420, SRIM_040735 |
|  | Cyanobacteria | | SRIM_040820, SRIM_035205, SRIM_028130, SRIM_026575, SRIM_025945, SRIM_021105, SRIM_014025, SRIM_009560, SRIM_040830, SRIM_031925, SRIM_028145, SRIM_018970, SRIM_014665, SRIM_009610, SRIM_003495, SRIM_028135, SRIM_010935, SRIM_024090, SRIM_012540, SRIM_028875, SRIM_001445, SRIM_018390, SRIM_020510, SRIM_020765, SRIM_039505, SRIM_023135, SRIM_025905, SRIM_031745, SRIM_034255, SRIM_036415 |
|  | Tenericutes | | SRIM_030485, SRIM_025945, SRIM_028145, SRIM_009610, SRIM_028435, SRIM_028135, SRIM_004055, SRIM_034035, SRIM_018390, SRIM_020510, SRIM_020510, SRIM_036415 |
|  | Spirochaetes | | SRIM_025945, SRIM_018390 |
|  | *Deinococcus-Thermus | | SRIM_029990, SRIM_025945, SRIM_025275, SRIM_009560, SRIM_004240, SRIM_028145, SRIM_005835, SRIM_028135, SRIM_020390, SRIM_018390, SRIM_031745 |
| Archea | Crenarchaeota | | SRIM_040820, SRIM_035205, SRIM_031215, SRIM_030485, SRIM_025945, SRIM_025275, SRIM_024130, SRIM_014025, SRIM_007730, SRIM_004240, SRIM_040830, SRIM_028145, SRIM_023470, SRIM_018970, SRIM_014665, SRIM_005835, SRIM_003495, SRIM_028135, SRIM_035545, SRIM_004055, SRIM_034035, SRIM_012540, SRIM_028875, SRIM_001445, SRIM_007705, SRIM_016865, SRIM_018390, SRIM_020510, SRIM_023135, SRIM_039505, SRIM_023985, SRIM_025905, SRIM_027005, SRIM_031745, SRIM_034255, SRIM_040360 |
|  | Euryarchaeota | | SRIM_025945, SRIM_023350, SRIM_031925, SRIM_028145, SRIM_014665, SRIM_028135, SRIM_023945, SRIM_018390, SRIM_020510, SRIM_031745 |
| **Cellular processes and signaling** | | | |
| Gram + | | Firmicutes | SRIM_030215, SRIM_027135, SRIM_023630, SRIM_018645, SRIM_006810, SRIM_027850, SRIM_028240, SRIM_004940, SRIM_015590, SRIM_006840, SRIM_012680, SRIM_024465, SRIM_010835, SRIM_026930, SRIM_018295, SRIM_023050, SRIM_022025, SRIM_020800, SRIM_017990, SRIM_014215, SRIM_001820, SRIM_025105, SRIM_021000, SRIM_020795, SRIM_018190, SRIM_010555, SRIM_007050, SRIM_001815, SRIM_026145, SRIM_031465, SRIM_023435, SRIM_010400, SRIM_010385, SRIM_010375, SRIM_020990, SRIM_018315, SRIM_001300, SRIM_002990, SRIM_003945, SRIM_004755, SRIM_007275, SRIM_020400, SRIM_021190, SRIM_028105, SRIM_028665, SRIM_008500, SRIM_015580, SRIM_030205 |
|  |  | Actinobacteria | SRIM_030215, SRIM_023630, SRIM_018645, SRIM_017755, SRIM_006810, SRIM_027850, SRIM_021195, SRIM_028240, SRIM_004940, SRIM_015590, SRIM_007590, SRIM_006840, SRIM_012680, SRIM_024465, SRIM_010835, SRIM_026930, SRIM_018295, SRIM_023050, SRIM_022025, SRIM_020800, SRIM_017990, SRIM_014215, SRIM_025105, SRIM_021000, SRIM_020985, SRIM_020795, SRIM_018190, SRIM_010555, SRIM_007050, SRIM_001815, SRIM_005235, SRIM_026145, SRIM_031465, SRIM_023435, SRIM_010400, SRIM_010385, SRIM_010375, SRIM_020990, SRIM_018315, SRIM_020980, SRIM_014315, SRIM_002105, SRIM_034905, SRIM_029210, SRIM_026815, SRIM_014620, SRIM_001300, SRIM_002990, SRIM_003945, SRIM_004755, SRIM_007275, SRIM_014230, SRIM_020400, SRIM_021150, SRIM_028665, SRIM_030205, SRIM_008500, SRIM_015580, SRIM_021190 |
| Gram - | | Proteobacteria | SRIM_030215, SRIM_023630, SRIM_018645, SRIM_017755, SRIM_006810, SRIM_027850, SRIM_028240, SRIM_004940, SRIM_026930, SRIM_018295, SRIM_023050, SRIM_022025, SRIM_020800, SRIM_017990, SRIM_014215, SRIM_001820, SRIM_025105, SRIM_021000, SRIM_020985, SRIM_020795, SRIM_018190, SRIM_010555, SRIM_007050, SRIM_001815, SRIM_021670, SRIM_023435, SRIM_010400, SRIM_014315, SRIM_029210, SRIM_002990, SRIM_003945, SRIM_004755, SRIM_007275, SRIM_021190, SRIM_028105, SRIM_028665, SRIM_015580, SRIM_030205 |
|  |  | Cyanobacteria | SRIM_023630, SRIM_018645, SRIM_027850, SRIM_028240, SRIM_004940, SRIM_010835, SRIM_018295, SRIM_023050, SRIM_022025, SRIM_020800, SRIM_017990, SRIM_014215, SRIM_001820, SRIM_025105, SRIM_021000, SRIM_020985, SRIM_020795, SRIM_018190, SRIM_010555, SRIM_007050, SRIM_005235, SRIM_031465, SRIM_023435, SRIM_010385, SRIM_029210, SRIM_002990, SRIM_003945, SRIM_004755, SRIM_007275, SRIM_028665, SRIM_008500, SRIM_015580, SRIM_021150, SRIM_030205 |
|  |  | Tenericutes | SRIM_028240, SRIM_004940, SRIM_018295, SRIM_020985, SRIM_023435, SRIM_003945, SRIM_021150, SRIM_028665 |
|  |  | Spirochaetes | SRIM_023050, SRIM_020800, SRIM_017990, SRIM_025105, SRIM_021000, SRIM_020795, SRIM_018190, SRIM_010555, SRIM_001300, SRIM_004755, SRIM_007275, SRIM_030205 |
|  |  | *Deinococcus-Thermus | SRIM_023630, SRIM_018295, SRIM_020800, SRIM_017990, SRIM_014215, SRIM_025105, SRIM_021000, SRIM_020795, SRIM_018190, SRIM_010555, SRIM_007050, SRIM_010400, SRIM_002990, SRIM_007275, SRIM_015580, SRIM_021190, SRIM_030205 |
|  |  | Thermotogae | SRIM_007275, SRIM_030205 |
| Archea | | Crenarchaeota | SRIM_023630, SRIM_018645, SRIM_028240, SRIM_004940, SRIM_007585, SRIM_026930, SRIM_020800, SRIM_017990, SRIM_025105, SRIM_021000, SRIM_020795, SRIM_018190, SRIM_010555, SRIM_007050, SRIM_023435, SRIM_003945, SRIM_021150 |
|  |  | Euryarchaeota | SRIM_027850, SRIM_028240, SRIM_004940, SRIM_023435, SRIM_029210, SRIM_003945, SRIM_004755, SRIM_007275, SRIM_030205 |
| **Information storage and processing** | | | |
| Gram + | | Firmicutes | SRIM_029570, SRIM_029330, SRIM_015460, SRIM_028075, SRIM_018615, SRIM_030245, SRIM_029960, SRIM_007210, SRIM_015925, SRIM_039930, SRIM_002125, SRIM_021285, SRIM_021640 |
|  |  | Actinobacteria | SRIM_029570, SRIM_029330, SRIM_015460, SRIM_028075, SRIM_018615, SRIM_018535, SRIM_019050, SRIM_029980, SRIM_029960, SRIM_024360, SRIM_015925, SRIM_021055, SRIM_039930, SRIM_006655, SRIM_015385, SRIM_021640 |
| Gram - | | Proteobacteria | SRIM_029570, SRIM_029330, SRIM_015460, SRIM_028075, SRIM_018615, SRIM_018535, SRIM_007455, SRIM_019050, SRIM_030245, SRIM_029980, SRIM_029960, SRIM_003375, SRIM_019760, SRIM_039930, SRIM_002125, SRIM_021640, SRIM_021285, SRIM_029730 |
|  |  | Cyanobacteria | SRIM_029330, SRIM_015460, SRIM_028075, SRIM_018615, SRIM_030245, SRIM_021285 |
|  |  | Tenericutes | SRIM_018615, SRIM_029960, SRIM_032170 |
|  |  | Spirochaetes |  |
|  |  | *Deinococcus-Thermus | SRIM_018615, SRIM_003375 |
| Archea | | Crenarchaeota | SRIM_018615 |
|  |  | Euryarchaeota | SRIM_018615 |
| **Unknown** | | | |
| Gram + | | Firmicutes | SRIM_006750, SRIM_022055, SRIM_009140, SRIM_013125, SRIM_028925 |
|  |  | Actinobacteria | SRIM_005995, SRIM_030020, SRIM_009140, SRIM_019835, SRIM_021080, SRIM_019790, SRIM_018745, SRIM_016410, SRIM_011000, SRIM_008900, SRIM_039385, SRIM_024125, SRIM_039670, SRIM_031615, SRIM_028925 |
| Gram - | | Proteobacteria | SRIM_022055, SRIM_013715, SRIM_018745, SRIM_028925 |
|  |  | Cyanobacteria | SRIM_018745, SRIM_028925 |
|  |  | Tenericutes |  |
|  |  | Spirochaetes |  |
|  |  | *Deinococcus-Thermus |  |
|  |  | Thermotogae |  |
| Archea | | Crenarchaeota | SRIM_028925 |
|  |  | Euryarchaeota |  |

* These bacteria have thick cell walls that give them Gram^+^ stains. Since they have a second membrane they are closer in structure to Gram^-^ bacteria, thus they were classified as Gram^-^
